# Multi-kingdom microbiota analyses identify bacterial–fungal interactions and biomarkers of colorectal cancer across cohorts

**DOI:** 10.1038/s41564-021-01030-7

**Published:** 2022-01-27

**Authors:** Ning-Ning Liu, Na Jiao, Jing-Cong Tan, Ziliang Wang, Dingfeng Wu, An-Jun Wang, Jie Chen, Liwen Tao, Chenfen Zhou, Wenjie Fang, Io Hong Cheong, Weihua Pan, Wanqing Liao, Zisis Kozlakidis, Christopher Heeschen, Geromy G. Moore, Lixin Zhu, Xingdong Chen, Guoqing Zhang, Ruixin Zhu, Hui Wang

**Affiliations:** 1 State Key Laboratory of Oncogenes and Related Genes, Center for Single-Cell Omics, School of Public Health, Shanghai Jiao Tong University School of Medicine, Shanghai, China; 2 National Clinical Research Center for Child Health, the Children’s Hospital, Zhejiang University School of Medicine, Hangzhou, China; 3 Guangdong Institute of Gastroenterology, Guangdong Provincial Key Laboratory of Colorectal and Pelvic Floor Diseases, Department of Colorectal Surgery, the Sixth Affiliated Hospital, Sun Yat-sen University, Guangzhou, China; 4 Research Institute, GloriousMed Clinical Laboratory Co., Ltd., Shanghai, China; 5 Clinical Medicine Transformation Center and Office of Academic Research, Shanghai Hospital of Traditional Chinese Medicine Affiliated to Shanghai University of traditional Chinese Medicine, Shanghai, China; 6 Department of Gastroenterology, The Shanghai Tenth People’s Hospital, Department of Bioinformatics, School of Life Sciences and Technology, Tongji University, Shanghai, China; 7 Bioinformatics Division, GloriousMed Clinical Laboratory Co., Ltd, Shanghai, China; 8 Chinese Academy of Sciences Key Laboratory of Computational Biology, Bio-Med Big Data Center, Shanghai Institute of Nutrition and Health, University of the Chinese Academy of Sciences, Chinese Academy of Sciences, Shanghai, China; 9 Shanghai Key Laboratory of Molecular Medical Mycology, Department of Dermatology, Shanghai Changzheng Hospital, Second Military Medical University, Shanghai, China; 10 Laboratory Services and Biobanking, International Agency for Research on Cancer, World Health Organization, Lyon, France; 11 United States Department of Agriculture, Agricultural Research Service, Southern Regional Research Center, New Orleans, LA USA; 12 State Key Laboratory of Genetic Engineering, Human Phenome Institute and School of Life Sciences, Fudan University, Shanghai, China; 13 Fudan University Taizhou Institute of Health Sciences, Taizhou, China

**Keywords:** Microbiome, Computational models

## Abstract

Despite recent progress in our understanding of the association between the gut microbiome and colorectal cancer (CRC), multi-kingdom gut microbiome dysbiosis in CRC across cohorts is unexplored. We investigated four-kingdom microbiota alterations using CRC metagenomic datasets of 1,368 samples from 8 distinct geographical cohorts. Integrated analysis identified 20 archaeal, 27 bacterial, 20 fungal and 21 viral species for each single-kingdom diagnostic model. However, our data revealed superior diagnostic accuracy for models constructed with multi-kingdom markers, in particular the addition of fungal species. Specifically, 16 multi-kingdom markers including 11 bacterial, 4 fungal and 1 archaeal feature, achieved good performance in diagnosing patients with CRC (area under the receiver operating characteristic curve (AUROC) = 0.83) and maintained accuracy across 3 independent cohorts. Coabundance analysis of the ecological network revealed associations between bacterial and fungal species, such as *Talaromyces islandicus* and *Clostridium saccharobutylicum*. Using metagenome shotgun sequencing data, the predictive power of the microbial functional potential was explored and elevated D-amino acid metabolism and butanoate metabolism were observed in CRC. Interestingly, the diagnostic model based on functional EggNOG genes achieved high accuracy (AUROC = 0.86). Collectively, our findings uncovered CRC-associated microbiota common across cohorts and demonstrate the applicability of multi-kingdom and functional markers as CRC diagnostic tools and, potentially, as therapeutic targets for the treatment of CRC.

## Abstract (web summary)

Analysis of bacterial, archaeal, fungal and viral species in the gut microbiome of patients with colorectal cancer identified cross-kingdom interactions and multi-kingdom markers of disease.

## Main

As the second leading cause of cancer-related deaths worldwide, colorectal cancer (CRC) accounts for approximately 900,000 deaths annually^1–5^. Incidence is still increasing worldwide, largely due to lifestyle and environmental factors, which severely affect the CRC-associated gut microbiota^6,7^. A growing body of literature demonstrates the dysregulated microbial structure in individuals with CRC, especially for bacterial microbiota^3,8–11^. For example, putatively procarcinogenic bacteria, including *Fusobacterium nucleatum*, *Escherichia coli*, enterotoxigenic *Bacteroides fragilis* and *Peptostreptococcus* spp., are increased in the faeces from patients with CRC. In contrast, protective genera, such as *Clostridium*, *Roseburia*, *Faecalibacterium* and *Bifidobacterium* are deminished^9,12–14^.

However, non-bacterial microorganisms including fungi, archaea and viruses, were also altered in CRC, adding further complexity to CRC microbiome association studies^2,15^. Nakatsu et al.^16^ found increased diversity of gut viromes in patients with CRC and revealed the contribution of an altered fungal ecology and co-occurrence interactions between fungi and bacteria to CRC^17^. Coker et al.^18^ also demonstrated the potential use of halophilic archaea in CRC diagnosis and the contribution of interactions between CRC-enriched archaea and bacteria in colon carcinogenesis.

The above studies highlighted the important roles of multi-kingdom microorganisms in CRC microbiota dysbiosis, while their fluctuations across different and large-scale populations are unexplored^16–19^. Moreover, discrepant results have been published^20^, which may be related to different biological factors and inconsistent standards for metagenomic data generation and processing. Recently, several attempts were made to identify the core CRC-associated bacterial microbiome signatures by meta-analysis using published shotgun metagenomic sequencing datasets. These studies provide an unbiased evaluation of CRC-associated bacterial microbiomes across multiple cohorts^12,13,21^. However, they did not determine the consistency, or potential inconsistency, of a multi-kingdom microbiome across different populations in CRC.

Although traditional screenings reportedly aid in reducing CRC incidence and mortality, the high false positive rate of faecal occult blood or faecal immunochemical tests, as well as the risk and expense of gold standard colonoscopy, represent relevant concerns^22–26^. Thus, major efforts have been made to explore complementary strategies of CRC diagnosis, including the potential application of gut microbiomes as non-invasive biomarkers for CRC. However, the clinical implementation of microbial-based diagnostic tools is challenging due to the heterogeneity of patient populations and associated high costs. For the time being, diagnostic tools based on multi-kingdom microbiome analysis should be used as a supplement to traditional CRC screening methods. Therefore, cross-cohort, multi-kingdom studies are urgently needed to provide integrated and robust assessment of CRC and multi-kingdom microbiome association.

In this study, we performed a comprehensive analysis of metagenomic datasets to assess the collective predictability of single- and multi-kingdom microbiota across eight distinct geographical cohorts. We took advantage of meta-analysis methods with a uniform pipeline for heterogeneity (MMUPHin) and a machine learning algorithm to identify multi-kingdom microbial markers. Our study demonstrates that diagnostic models with multi-kingdom markers perform better than models based on single-kingdom markers. A minimal panel with 16 multi-kingdom microbial features diagnosed patients with CRC with an area under the receiver operating characteristic curve (AUROC) of 0.83 and maintained accuracy across 3 independent cohorts. Moreover, exploration of the metagenomic functions in CRC highlighted the elevated metabolic potentials of D-amino acid and butanoate metabolism. Interestingly, the diagnostic model based on functional genes achieved high accuracy (AUROC = 0.86). Collectively, these findings uncover common and comprehensive CRC-associated microbiota and reveal the potential of multi-kingdom and functional markers as powerful CRC diagnosis tools and, potentially, as therapeutic targets for the treatment of CRC.

## Results

### Characterization of multi-cohort CRC and processing of shotgun metagenomic sequencing data

**Fig. 1:**
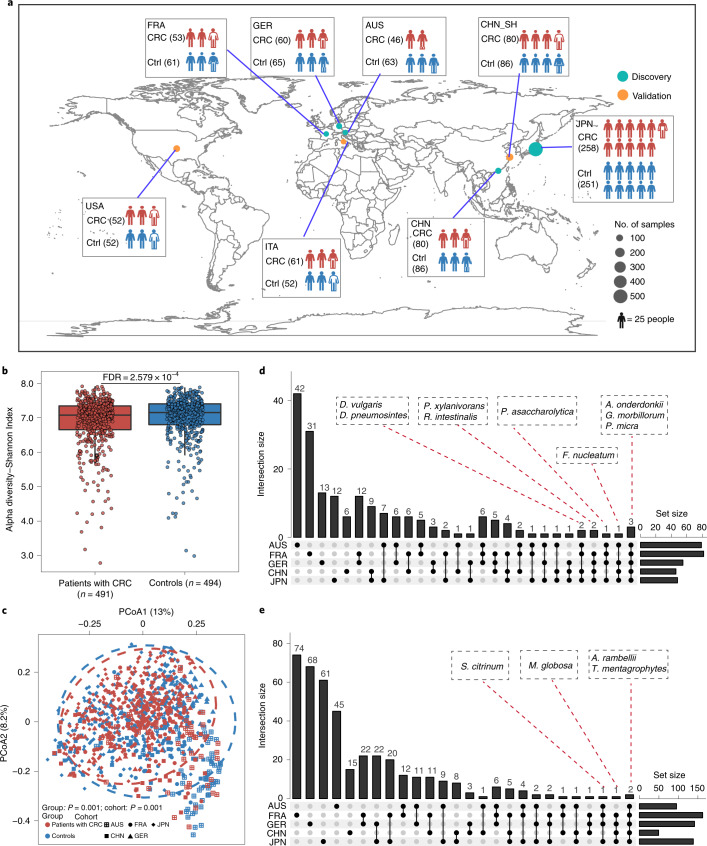
Overview of the patient populations with CRC included in this study and their associated gut microbiome compositions. **a**, Global map representing a total of 1,368 samples from 8 patient populations with faecal shotgun metagenomic data. The discovery data populations included Austria (AUS, PRJEB7774), France (FRA, PRJEB6070), Germany (GER, PRJEB27928), China (CHN_HK, PRJEB10878) and Japan (JPN, PRJDB4176). The validation data populations included the United States (USA, PRJEB12449), Italy (ITA, SRP136711) and China (CHN_SH, in-house). The numbers in brackets represents sample size. Details are shown in Supplementary Data 1. **b**, Alpha diversity measured by Shannon index of patients with CRC (red, *n* = 491) and control individuals (blue, *n* = 494). Adjusted *P* value (FDR = 2.579 × 10^−4^, two-sided test) was calculated by MMUPHin. Data are shown via the interquartile ranges (IQRs) with the median as a black horizontal line and the whiskers extending up to the most extreme points within 1.5× the IQR; outliers are represented as dots. **c**, Principal coordinate analysis (PCoA) of samples from all five cohorts based on Bray–Curtis distance, which shows that microbial composition was different between groups (*P* = 0.001) and cohorts (*P* = 0.001). *P* values of beta diversity based on Bray–Curtis distance were calculated with PERMANOVA by 999 permutations (two-sided test). The group is colour-coded and the cohort is indicated by different shapes. **d**, UpSet plot showing the number of differential bacterial species identified via MaAsLin2 in each population and shared by combinations of datasets. The number above each column represents the size of differential species. The set size on the right represents the number of differential species in each cohort and the connected dots represent the common differential species across connected cohorts. **e**, UpSet plot showing the number of differential fungal species identified via MaAsLin2 in each population and shared by combinations of datasets. The number above each column represents the size of differential species. The set size on the right represents the number of differential species in each cohort and the connected dots represent the common differential species across connected cohorts. Source data

We collected multi-cohort CRC metagenomic data from 1,368 samples, consisting of population data from 7 publicly available cohorts and one new Chinese (CHN_SH) cohort (Supplementary Data 1 and Fig. 1a). To identify reproducible microbial markers for diagnosing patients with CRC, the discovery dataset consisted of samples with broader geographical heterogeneity and genetic background, including 491 individuals with CRC and 494 tumour-free controls across 5 countries (Austria, France, Germany, China and Japan) (Fig. 1a). Accordingly, the independent validation dataset consisted of 193 patients with CRC and 190 controls covering 3 countries (China, Italy and the USA) (Fig. 1a). To reduce technical bias in the bioinformatics analysis, all raw shotgun sequencing data were processed consistently (Supplementary Data 2).

### Integrated analysis of CRC-associated microbial species in four kingdoms

**Extended Data Fig. 1:**
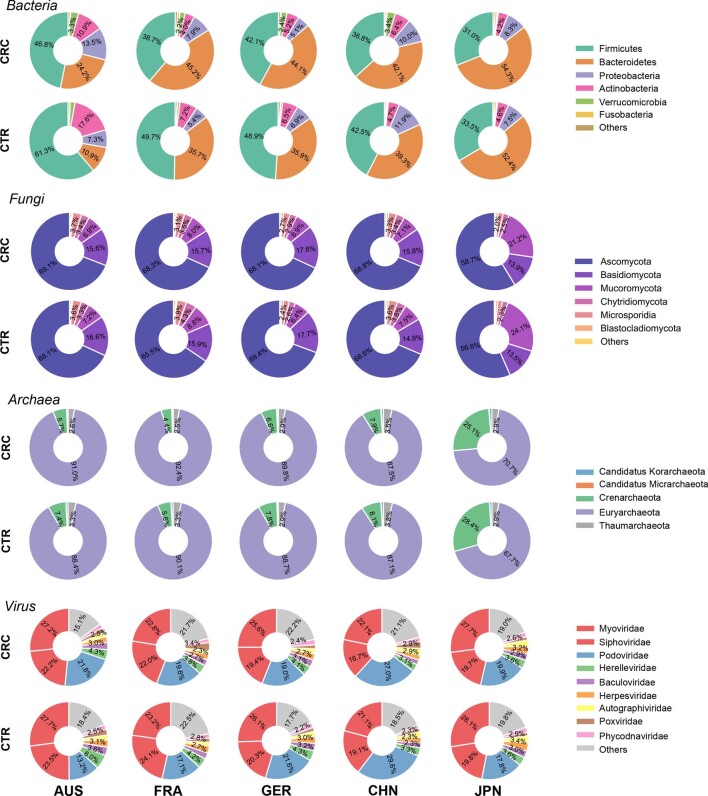
Overview of microbial composition in four kingdoms across populations. Microbial composition of four kingdoms in CRC and control (CTR) group, respectively. Composition of bacteria, fungi and archaea was shown at phylum level and composition of virus was shown at family level. Only the abundant phyla are shown in the pie chart and the rare family are summed into others. Source data

We first assessed changes in alpha diversity of patients with CRC and healthy controls. Decreased microbial alpha diversity assessed by the Shannon Index was observed for CRC (false discovery rate (FDR) = 2.579 × 10^−4^; Fig. 1b). Notably, differences in beta diversity varied not only according to disease status (*P* = 0.001, Fig. 1c) but also across cohorts (*P* = 0.001; Fig. 1c). Regarding microbial composition, we found different microbial alterations across all four kingdoms for the CRC samples at the phylum level (Supplementary Discussion, Extended Data Fig. 1 and Supplementary Data 3).

**Extended Data Fig. 2:**
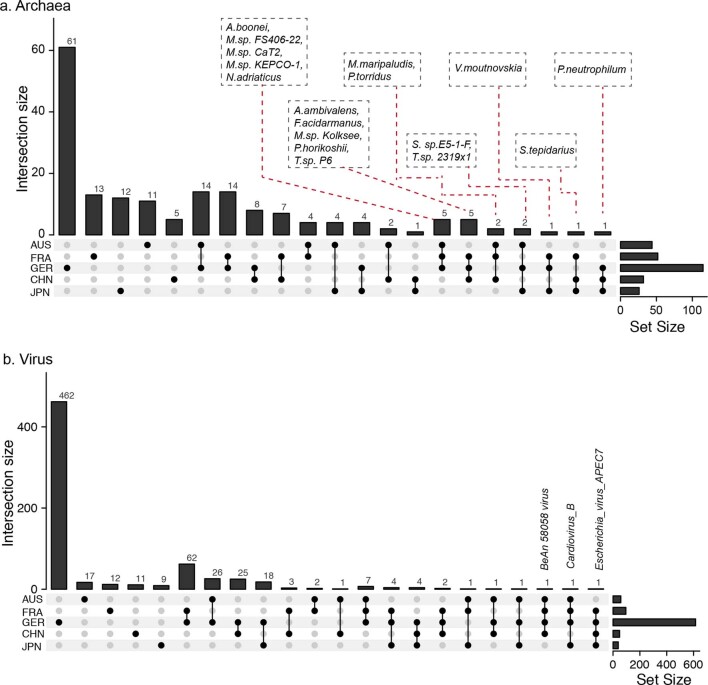
Differential species of archaea and virus among populations. **a**, UpSet plot showing the number of differential archaea species identified via MaAsLin2 in each population and shared by combinations of datasets. The number on top of each column represents the size of differential species. The set size on the right represents the number of differential species in each cohort and connected dots represent the common differential species across connected cohorts. **b**, UpSet plot showing the number of differential viral species identified via MaAsLin2 in each population and shared by combinations of datasets. The number on top of each column represents the size of differential species. The set size on the right represents the number of differential species in each cohort and connected dots represent the common differential species across connected cohorts. Source data

**Extended Data Fig. 3:**
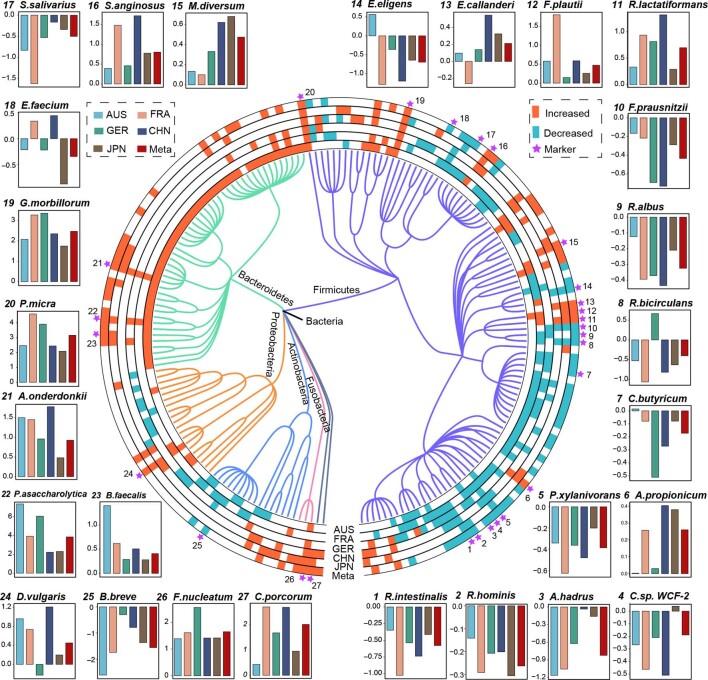
Differential bacterial species across populations and prediction performances of models constructed with each single-kingdom features. Phylogenetic tree showing the union of different bacterial species (88 in total), grouped by the phyla grouped in the phyla Actinobacteria, Basidiomycota, Proteobacteria, Bacteroidetes, Firmicutes and so on. The outer circles are marked for significant differential species (*p* < 0.05, two-sided test) in each population and the meta-analysis results identified via MMUPHin with orange for increased species and green for decreased species. Species marked with purple stars were features selected in the classification model. Bar plots show the abundances fold change (FC) normalized by log of marker features in each population. The number represents the marker number marked with star. Color represents population and red bars are FC of all subjects in CRC and CTR. Source data

To identify specific microbial markers for potential CRC diagnosis, we next examined species composition. Although differential microbial species varied greatly in different cohorts, some species with consistent alterations were identified, such as the bacteria *Alistipes onderdonkii*, *Parvimonas micra* and *Gemella morbillorum* (Fig. 1d) and the fungi *Aspergillus rambellii* and *Trichophyton mentagrophytes* (Fig. 1e). However, the differential species of archaea (Extended Data Fig. 2a) and viruses (Extended Data Fig. 2b) displayed substantial variations without consistent differential species across cohorts. These findings necessitate an integrated analysis to identify universal microbial markers for CRC. Our analysis identified 88 bacterial, 108 fungal, 38 archaeal and 115 viral species, with differential abundances between individuals with CRC and controls, respectively (*P* < 0.05; Supplementary Data 4). Consistent with reported bacterial alterations in CRC, 48 bacterial species with elevated abundances in patients with CRC were identified (Extended Data Fig. 3), including the widely reported *F. nucleatum*, *P. micra*, *Porphyromonas asaccharolytica*, *Desulfovibrio desulfuricans* and *Akkermansia muciniphila*. In particular, protective species from butyrate-producing bacteria, such as *Clostridium butyricum*, *Roseburia intestinalis* and *Butyrivibrio fibrisolvens*, were decreased in patients with CRC compared to controls.

**Fig. 2:**
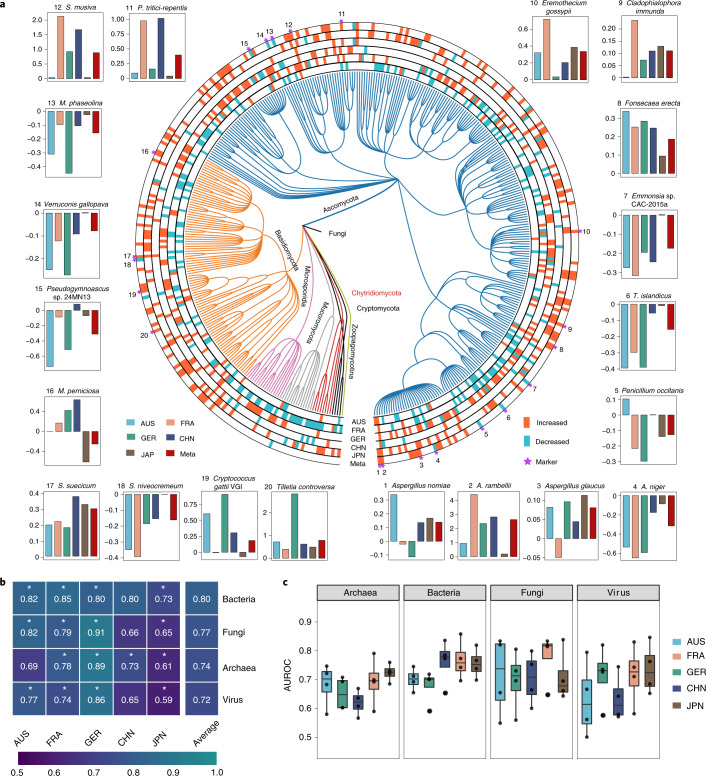
Differential species across populations and prediction performances of models constructed with each single-kingdom features. **a**, Phylogenetic tree showing the union of differential fungal species (231 in total), grouped by the phyla Ascomycota, Basidiomycota, Chytridiomycota, Cryptomycota, Microsporidia, Mucoromycota and Zoopagomycotina. The outer circles are marked for significant differential species (*P* < 0.05, two-sided test) in each cohort; the meta-analysis results were identified via MMUPHin (meta-ring) with orange for increased species and green for decreased species. Species marked with purple stars represent features selected in the classification model. The bar plots show the abundance fold change normalized by the log of marker features in each population. The number represents the marker number, which is marked with a star. The colour represents the population and the red bars are the fold change of all individuals in the CRC and control groups. **b**, The heatmap shows the AUROC values of models built with single-kingdom features in each cohort. The values refer to the average value of the 20× fivefold cross-validation. The asterisk represents the significance of models assessed with 1,000 permutations (two-sided test). *P = 0.001. **c**, Box plots showing the cohort-to-cohort AUROC values of the models using the features of each kingdom. Data are shown via the IQRs, with the median as a black horizontal line and the whiskers extending up to the most extreme points within the 1.5× the IQR (*n* = 4). Source data

**Extended Data Fig. 4:**
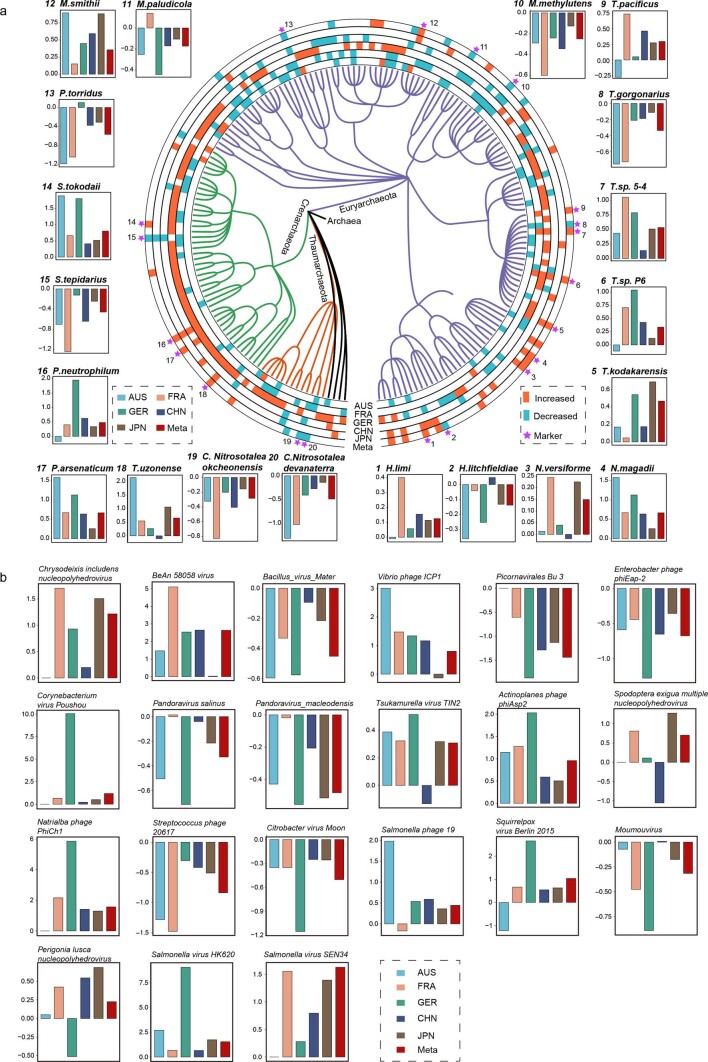
Differential archaeal species across populations and prediction performances of models constructed with each single-kingdom features. a, Phylogenetic tree showing the union of differential archaeal species (38 in total), grouped by the phyla grouped in the phyla Crenarchaeota, Euryarchaeota, Thaumarchaeota and Candidatus Korarchaeota. The outer circles are marked for significant differential species (p < 0.05, two-sided test) in each population and the meta-analysis results identified via MMUPHin with orange for increased species and green for decreased species. Species marked with purple stars were features selected in the classification model. Bar plots show the abundances fold change (FC) normalized by log of marker features in each population. The number represents the marker number marked with star. Color represents population and red bars are FC of all subjects in CRC and CTR. b, Bar plots show the abundances fold change (FC) normalized by log of viral marker features in each population. Color represents population and red bars are FC of all subjects in CRC and CTR. Source data

Apart from gut bacteria, emerging studies suggest the importance of other microbial kingdoms in gastrointestinal disease^17,27^. Intriguingly, the abundance of 93 out of 108 fungal species was increased in patients with CRC compared to controls (Fig. 2a), including *Candida pseudohaemulonis*, *Aspergillus ochraceoroseus*, *A. rambellii* and *Malassezia globosa*. In contrast, the abundances of *Aspergillus niger*, *Macrophomina phaseolina*, *Talaromyces islandicus* and *Sistotremastrum niveocremeum* were decreased in patients with CRC. Moreover, we also identified 38 archaeal species and 133 viral species with significantly differential abundances between patients with CRC and controls (Supplementary Discussion, Supplementary Data 4 and Extended Data Fig. 4).

### Single microbial kingdom markers for CRC diagnosis

**Extended Data Fig. 5:**
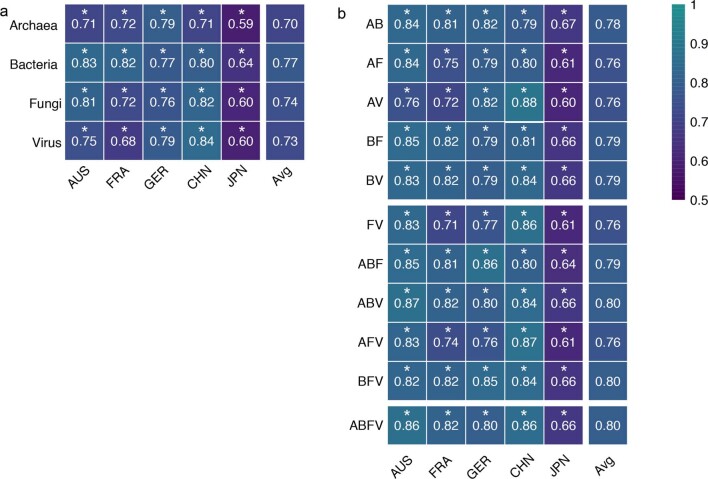
Performance of LOCO analysis in single- and multi-kingdom models. The AUROC values of LOCO analysis in single-kingdom **(a)** and multi-kingdom **(b)** models. The asterisk represents the significance of models assessed with 1000 permutations (two-sided test). *: *p* = 0.001. A: Archaea; B: Bacteria; F: Fungi and V: Virus. Source data

Recently, bacterial markers for CRC diagnosis have achieved satisfactory accuracy^12,13^. However, the predictive value of archaea, fungi and viruses is underestimated, especially across different cohorts. Hence, this comprehensive analysis investigated potential microbial markers from different kingdoms for CRC diagnosis (Extended Data Fig. 5). Ultimately, we identified 20 fungal (Fig. 2a), 27 bacterial (Extended Data Fig. 3), 20 archaeal and 21 viral species (Extended Data Fig. 4) as important features including *T. islandicus*, *Sphaerulina musiva*, *A. rambellii*, *A. niger* from the fungi kingdom (Fig. 2a) and *F. nucleatum*, *P. micra* and *P. asaccharolytica* from the bacteria kingdom (Extended Data Fig. 3).

We next constructed fivefold cross-validation random forest models with features from each single kingdom. As expected, features from each kingdom showed capabilities for identifying patients with CRC (Fig. 2b). The extensively studied bacterial models displayed the strongest ability to detect CRC across all cohorts with an average score of the AUROC of 0.80, ranging from 0.73 to 0.85. In comparison, the model for fungal features showed the second highest score with an average AUROC of 0.77. The archaeal- (average AUROC = 0.74) and virus-based models (average AUROC = 0.73) displayed relatively inferior distinguishing capability. Notably, the diagnostic capabilities showed great variation across different geographical cohorts (Supplementary Discussion; Fig. 2b), suggesting distinct gut microbiome characteristics for these patients with CRC, most likely due to dietary differences^9^. Overall, our results highlight that apart from bacteria and fungi, archaea and viruses also represent potential markers for CRC diagnosis.

To evaluate whether the above features could be applied universally for CRC diagnosis and overcome geographical heterogeneity, we performed cohort-to-cohort transfer analysis and leave-one-cohort-out (LOCO) analysis as described previously^11^. Overall, the AUROC scores based on the cohort-to-cohort transfer analysis were slightly reduced compared to the cross-validation models, while the AUROC values of LOCO were increased compared to those from the cohort-to-cohort transfer analysis (Supplementary Discussion, Fig. 2c and Extended Data Fig. 5a), probably due to the larger size of the ‘training’ dataset. Collectively, our findings demonstrate that marker features from different kingdoms provide unbiased predictive capabilities for CRC diagnosis across various populations.

### Improved predictability based on combined multi-kingdom features

**Fig. 3:**
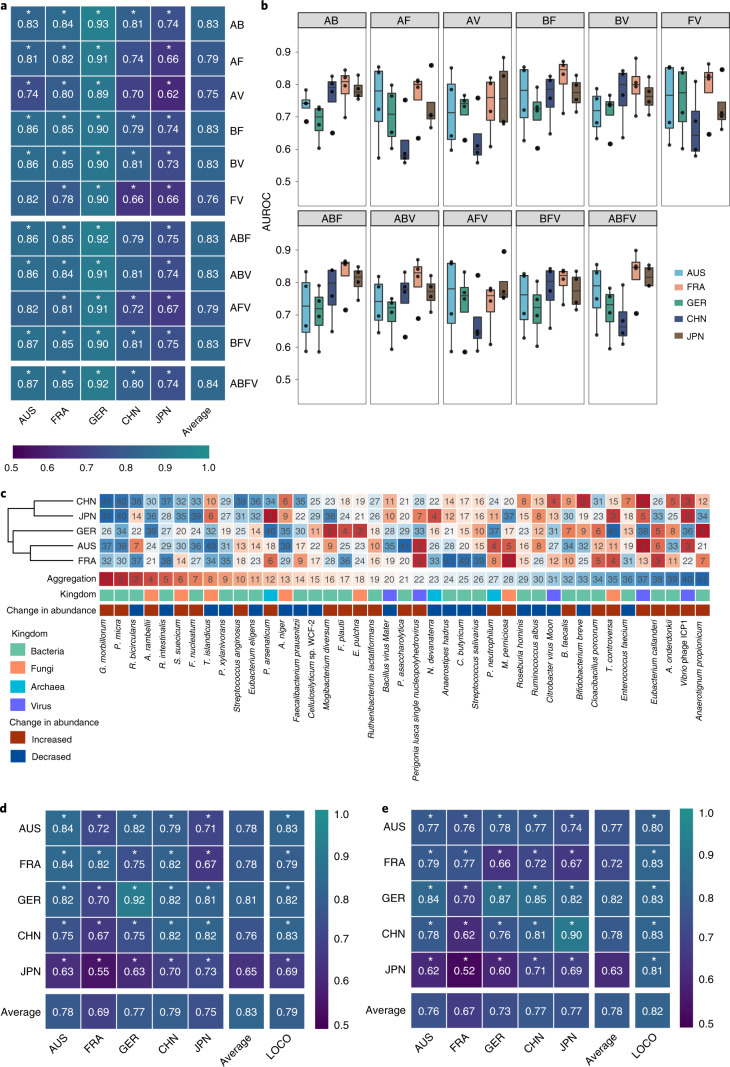
Performance of predictive models constructed with combined multi-kingdom features and the integrated importance of these essential features in each geographical cohort. **a**, Heatmap showing the AUROC values of the models built with multi-kingdom features in each cohort. The values refer to an average value of 20× repeated fivefold cross-validation. The asterisk represents the significance of models assessed with 1,000 permutations (two-sided test). *P = 0.001. A, Archaea; B, Bacteria; F, Fungi; V, Virus. **b**, Box plots showing the AUROC values of cohort-to-cohort transfer validation for the models using multi-kingdom features. Data are shown via the IQRs with the median as a black horizontal line and the whiskers extending up to the most extreme points within 1.5× the IQR (*n* = 4). **c**, Importance of each listed feature (belonging to the four-kingdom model) by the cross-validation of predictive performance for each population dataset as estimated using the internal random forest ‘Gini importance’ method. The ‘aggregation’ column shows the integrated ranks (using a rank aggregation algorithm) of listed markers within each cohort along with changes in abundance (differentials), with red indicating a species increase and blue indicating a species decrease in patients with CRC compared to controls. **d**, AUROC matrix of models built with the panel of 16 multi-kingdom features for CRC detection. Values on the diagonal refer to the average AUROC of 20× repeated fivefold stratified cross-validations. Values off the diagonal refer to the AUROCs obtained by training the model on the population of the corresponding row and applying it to the population of the corresponding column. The LOCO row refers to the performances obtained by training the model on the 16 microbial features using all but the population dataset of the corresponding column and applying it to the dataset of the corresponding column. The asterisk represents the significance of models assessed with 1,000 permutations (two-sided test). *P = 0.001. **e**, AUROC matrix of models built with the panel of 16 multi-kingdom features for CRC early detection. Values on the diagonal refer to the average AUROC of 20× repeated fivefold stratified cross-validations. Values off the diagonal refer to the AUROCs obtained by training the model on the population of the corresponding row and applying it to the population of the corresponding column. The LOCO row refers to the performances obtained by training the model on the 16 microbial features using all but the population dataset of the corresponding column and applying it to the dataset of the corresponding column. The asterisk represents the significance of models assessed with 1,000 permutations (two-sided test). *P = 0.001. Source data

Since all single-kingdom features displayed diagnostic potential for patients with CRC, we next explored the predictability of models combining individual multi-kingdom features. In line with our hypothesis, improved CRC detection was obtained by combining multi-kingdom features, suggesting an additive predictive value for the combination of different kingdom features. Compared to single-kingdom diagnostic models, the AUROC values of two-kingdom features were improved, ranging from 0.75 to 0.83 (Fig. 3a). Specifically, the cross-validation models combining bacteria- and archaea-based features (AB model) achieved an average AUROC of 0.83, which is higher than any single-kingdom model (AUROC = 0.80 for bacteria and 0.74 for archaea; Fig. 2b). The predictive value of models combining bacteria- and fungi-based features (BF model) also reached an average AUROC of 0.83. Specifically, the AUROC scores across different cohorts were 0.86 (AUS), 0.85 (FRA), 0.90 (GER), 0.79 (CHN) and 0.74 (JPN). Consistent improvements could be observed for other two-kingdom features. Furthermore, the transferability of multi-kingdom models was also enhanced (Supplementary Discussion, Figs. 2c and 3b and Extended Data Fig. 5b).

We then examined the predictive performance of models with three-kingdom feature combinations, which revealed no further improvements. All three-kingdom models achieved an average AUROC of 0.83, except for the archaea-fungi-virus (AFV) model (average AUROC = 0.79), which maintained the same accuracy as the best two-kingdom model, namely the BF (Fig. 3a). Consistently, the AUROCs for models based on four-kingdom features (ABFV model) only slightly improved (average AUROC = 0.84; Fig. 3a). Importantly, however, transferability for cohort-to-cohort (approximately 0.76 on average) and LOCO analysis (maximum AUROC = 0.80 for the ABV, BFV and ABFV models) was slightly improved for the three- and four-kingdom models, respectively (Extended Data Fig. 5b). In summary, the AUROCs of the multi-kingdom models significantly improved than those of the single-kingdom models (Supplementary Data 5).

Intrigued by our finding that the AUROCs did not markedly improve beyond the two-kingdom models with the addition of more markers, we sought to investigate the underlying reasons (Supplementary Discussion and Supplementary Data 6). We found that the ABFV models with a total 41 features contained 13 bacterial, 5 fungal, 1 archaeal and 1 viral marker as the top 20 features (Fig. 3c). Collectively, most of the predictable information was provided by bacterial and fungal markers. Thus, multi-kingdom models did not further enhance the performance of our diagnostic models.

### Identification of the best-performing panel of features derived from multi-kingdoms

Since models constructed with four-kingdom markers were the most effective for CRC diagnosis, particularly with respect to transferability between cohorts, we next aimed to identify the essential features of the four-kingdom models (Fig. 3c and Supplementary Data 7). First, bacterial species, that is, *G. morbillorum, P. micra*, *Ruminococcus bicirculans*, *R. intestinalis* and *F. nucleatum*^9,12,13^, were among the top five and seventh most important contributors to the predictive value of our four-kingdom models. Meanwhile, fungal species, such as *A. rambellii*, *Sistotremastrum suecicum*, *T. islandicus* and *A. niger*, were also identified as important features (4th, 6th, 8th and 13th rank, respectively). Three archaeal species (features), *Pyrobaculum arsenaticum*, *Nitrosotalea devanaterra* and *Pyrobaculum neutrophilum* ranked 12th, 23rd and 27th, respectively. Additionally, the butyrate-producing bacteria *Butyricimonas faecalis*, *Flavonifractor plautii*, *C. butyricum* and the fungal species (features) *Erysiphe pulchra* and *Moniliophthora perniciosa* also contributed to the predictability of the four-kingdom model. We also identified five viral species, although these achieved only lower ranks in our predictive model. Thus, our feature ranking analysis highlighted the need to combine features from multi-kingdoms, particularly those from the bacterial and fungal kingdoms, for maximized predictive value.

To identify a minimal panel of microbial markers, we successively added features according to their ranking (Gini importance). The average AUROC values maxed out after adding the top 16 features with an AUROC of 0.83, which included 11 bacterial features, 4 fungal features and 1 archaeal feature (Fig. 3d and Supplementary Data 8). This 16-feature multi-kingdom model also showed good performance in single populations. In all cohort models, CRC samples were identified with an accuracy above 0.82 except for the JPN cohort (AUROC = 0.73; Fig. 3d). The GER cohort showed the highest predictability with an AUROC of 0.92. The models also displayed an acceptable transferability across cohorts (Fig. 3d). Therefore, our analysis revealed a minimum panel of 16 features derived from bacteria, fungi and archaea kingdoms as a stool-based non-invasive tool for CRC diagnosis.

### Performance of 16 multi-kingdom marker panel for early CRC diagnosis

**Extended Data Fig. 6:**
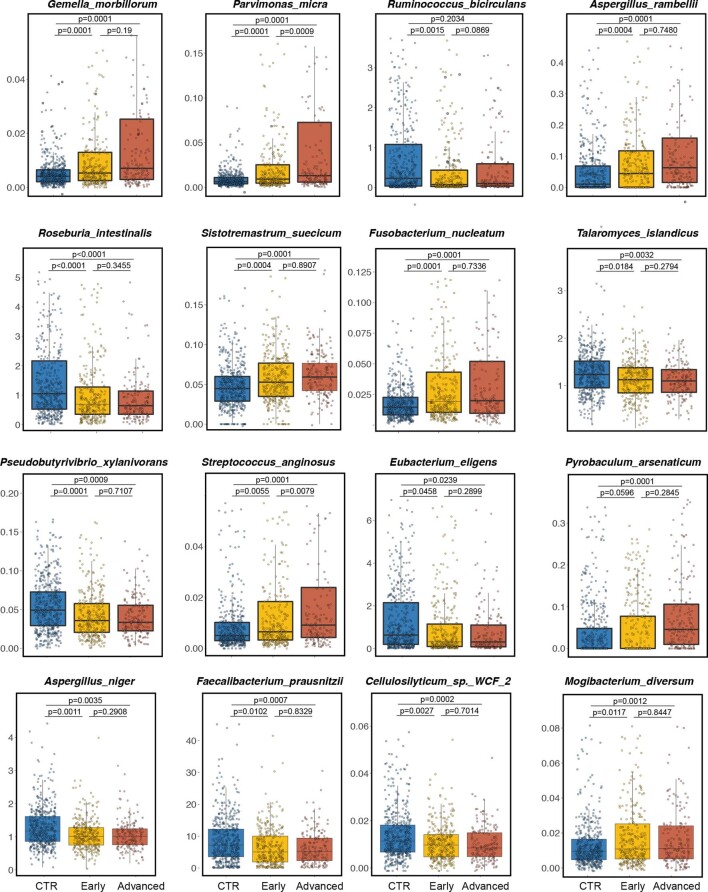
The abundance changes of the best panel of 16 multi-kingdom features changes in CTR (n = 494), early- stage CRC (n = 318) and advanced CRC (n = 173). The *p* values were calculated via MMUPHin (two-sided test). Data were showed via the interquartile ranges (IQRs) with the median as a black horizontal line and the whiskers extending up to the most extreme points within 1.5-fold IQR, and outliers are represented as dots. Source data

Diagnosing cancer at an early stage could significantly increase survival rates. Therefore, we investigated the predicative performance of a 16-marker multi-kingdom panel in early-stage (stage I and II) patients with CRC. Notably, the abundance of the 16 markers was significantly different not only between controls and patients with advanced CRC but also between controls and patients with early-stage CRC (Extended Data Fig. 6). This finding suggested the potential use of our marker panel for the early diagnosis of CRC. After adjusting for the unbalanced numbers of patients with early-stage CRC versus controls, our panel was able to distinguish patients with early-stage CRC from controls with an average AUROC of 0.78, which also showed excellent diagnostic transferability across cohorts with an average LOCO AUROC of 0.82 (Fig. 3e).

### Validation of the 16-marker multi-kingdom panel in independent cohorts

**Extended Data Fig. 7:**
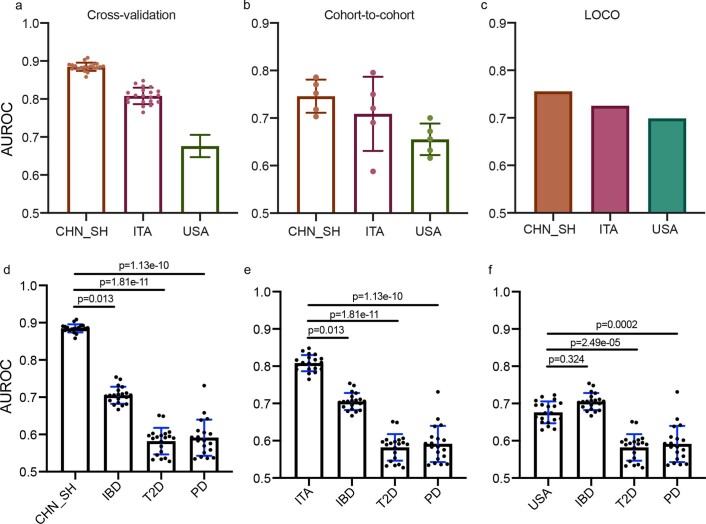
Validation of the multi-kingdom markers panel in independent cohorts and Specificity for CRC. The diagnostic accuracy based on the multi-kingdom markers for independent cohorts one from China, two from Italy and one from USA, is indicated by the (**a**) cross-validation, (**b**) cohort-to-cohort and (**c**) LOCO analysis, which was trained with cohorts in discovery datasets. Bar height for cross-validation corresponds to the average of 20 time repeats (the error bars indicate the s.d., n = 20), and the bar height for cohort-to-cohort analysis corresponds to the average of five models (the error bars indicate the mean ± SD, n = 5). The diagnostic accuracy based on the multi-kingdom markers for CRC in independent cohorts and non-CRC disease, including IBD, T2D and PD. The AUROC values of CHN_SH (**d**) and ITA (**e**) were significantly higher than that of non-CRC disease. The AUROC values of USA (**f**) were significantly higher than T2D and PD but with no difference with IBD. Bar height for analysis corresponds to the average of 20 times for five-fold cross-validation models (the error bars indicate the mean ± SD, n = 20). All the p value was adjusted by ‘Bonferroni’ via two-sided dunn test after Kruskal-Wallis test. Source data

To externally validate the predictive performance of our 16-marker multi-kingdom panel and avoid overoptimistic reporting of model accuracy, we analysed 3 independent datasets (Supplementary Data 1 and 2) from China (CHN_SH, 86 controls and 80 patients with CRC), Italy (ITA, 52 controls and 61 patients with CRC) and the United States (USA, 52 controls and 52 patients with CRC) (Extended Data Fig. 7a). The average AUROC of the cross-validation models was 0.88 for the CHN_SH cohort and 0.81 for the ITA cohort, respectively, while the AUROC was relatively lower for the USA (0.68 on average). The latter may be related to long-time frozen storage of samples (over 25 years). The average AUROC for the cohort-to-cohort analysis was relatively decreased (CHN_SH:0.75, ITA:0.71 and USA:0.66; Extended Data Fig. 7b), while the AUROCs for the LOCO analysis were slightly improved, ranging from 0.70 to 0.76 (Extended Data Fig. 7c). Altogether, this additional cohort analysis validated the robustness of our multi-kingdom marker panel across a total of eight cohorts from seven countries.

### Specificity of the CRC predictive models based on the multi-kingdom marker panel

In light of shared microbiota alterations across various diseases^28^, it is necessary to verify the disease specificity for the identified microbial biomarkers panel, thereby ensuring a low false positive rate for CRC diagnosis. For this purpose, several non-CRC disease datasets were assessed, including those from gastrointestinal disease (inflammatory bowel disease (IBD)) and non-gastrointestinal diseases (type 2 diabetes (T2D) and Parkinson’s disease (PD)) (Extended Data Fig. 7d–f). AUROC values were significantly lower for non-CRC diseases compared to our independent cohort of patients with CRC. Particularly, diagnostic accuracy sharply decreased in patients with IBD, T2D and PD compared to that in the CHN_SH and ITA cohorts with CRC. Overall, these results support the notion that our 16-biomarker multi-kingdom panel is highly specific to CRC.

### Alterations of the multi-kingdom coabundance network between patients with CRC and controls

**Fig. 4:**
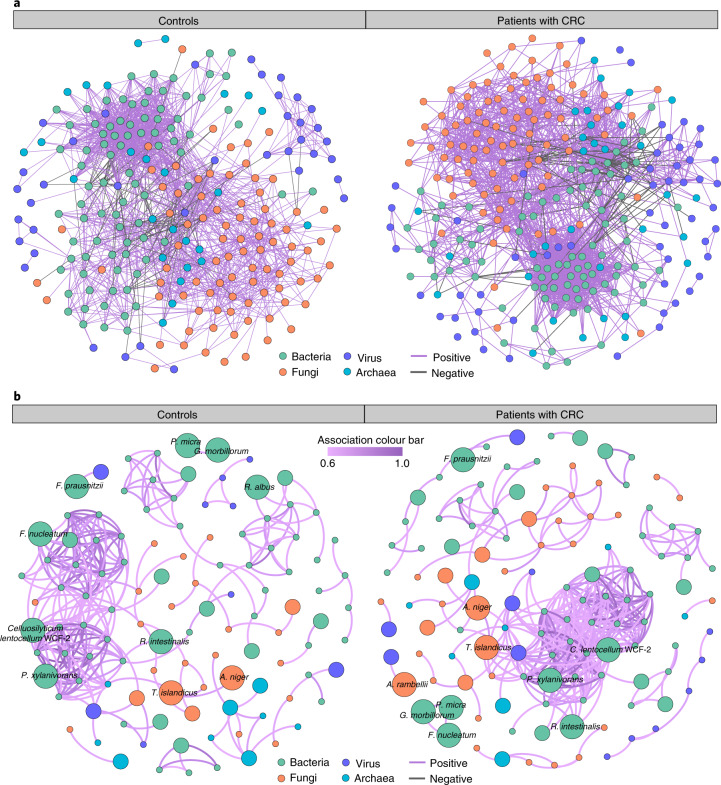
Coabundance correlations among multi-kingdom species in patients with CRC and controls. **a**, Coabundance networks involving combined differential species from all four kingdoms in the CRC and control samples. The colours of nodes indicate species from bacteria (green), fungi (orange), archaea (blue) and viruses (purple). Only significant (FDR < 0.00001, two-sided tests of 1,000 permutations) absolute correlations above 0.3 are shown, which are considered as fair correlations. The purple lines indicate positive species interactions; the grey lines indicate negative interactions. **b**, Moderate coabundance networks in controls and patients with CRC, with absolute correlations above 0.6 and with a significance cut-off of FDR < 0.00001 (two-sided tests of 1,000 permutations). The edges are coloured according to the magnitude of the association in the moderate networks as shown by the colour bar. Source data

To gain an insight into the potential interplay between multi-kingdom species and their potential role in CRC pathogenesis, we performed a coabundance association analysis based on the abundance of differential species. Generally, the ecological network of patients with CRC (272 species and 2,338 associations) was more complex compared to that of controls (236 species and 1,804 associations). Apart from intensive correlations between intrakingdom species, we found substantial associations between interkingdom species, especially between the bacteria and fungi kingdoms (Fig. 4a).

In addition, there were many increased correlations in the CRC network compared to the network in controls, including 1,161 intrakingdom and 706 interkingdom associations (Supplementary Data 9), which may play a role in CRC pathogenesis. In particular, emerged interkingdom interactions were discovered in the CRC microbiome, for example, correlations between the fungal markers *T. islandicus* and differential bacteria species, namely *Clostridium saccharobutylicum, Hungateiclostridium clariflavum, Clostridium baratii* and *Faecalibaculum rodentium*. Consistently, a similar pattern was also observed in networks with moderate associations (*r* > 0.6). Specifically, the network of controls consisted of 273 coabundance correlations among 112 species, while the CRC network contained 360 coabundance correlations among 120 species (Fig. 4b). Several markers belonging to the bacteria and fungi kingdoms were presented in the moderate networks, such as *G. morbillorum*, *P. micra*, *F. nucleatum*, *T. islandicus* and *A. rambellii*. However, there were only a few associations or weak correlations between diagnostic markers, probably due to their limited predictive value for the diagnostic models. Taken together, these findings suggest an important role for both intra- and interkingdom interactions in gut microbiota for CRC pathogenesis.

### Microbial functional alterations in CRC

Owing to the vast interindividual heterogeneity of the microbiota, it seems plausible that distinct strains in different individuals can trigger a similar pathology by utilizing common pathways. Therefore, targeting their wide-spanning metagenomic functions, rather than specific taxa, may represent a more effective strategy to investigate microbiome-mediated tumorigenesis in CRC.

**Fig. 5:**
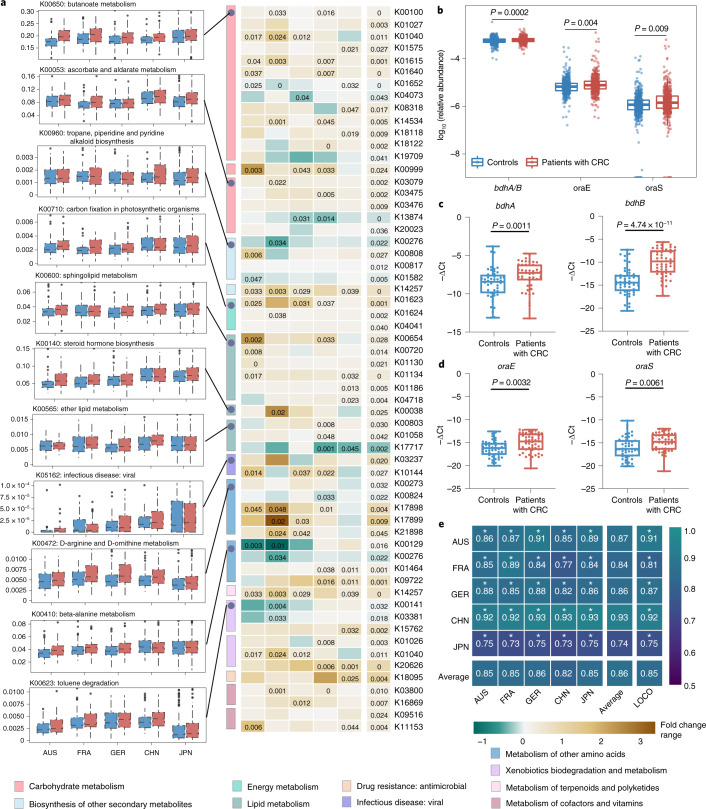
CRC-associated functional alterations and performance of models constructed with KO genes. **a**, The box plots (left) show the relative abundance of the pathway of controls (blue bar) and patients with CRC (red bar) in each cohort. The number of samples was AUS (patients with CRC = 46, controls = 63), FRA (patients with CRC = 53, controls = 61), GER (patients with CRC = 60, controls = 65), CHN (patients with CRC = 80, controls = 86), JPN (patients with CRC = 258, controls = 251), respectively. All box plots represent the 25th–75th percentile of the distribution; the median is shown as a thick line in the middle of the box; the whiskers extend up to the most extreme points within a 1.5× the IQR and outliers are represented as dots. The heatmap (centre) shows the integrated meta-analysis that identified significantly changed KO gene expression in each metabolic pathway examined across five geographical populations. The cell colour and intensity represent the generalized abundance fold change of KO genes. The significant differential KO gene (*P* < 0.05, two-sided test) was identified via MMUPHin. *P* values are shown in the cells. **b**, Normalized log abundance for the functional genes *bdhA/B* (K00100), *oraE* (K17898) and *oraS* (K17899) is compared between controls (n = 494) and patients with CRC (*n* = 491). Statistical significance was determined via MMUPHin with treating age, BMI and sex as covariates (two-sided test). **c**,**d**, Expression of *bdhA* and *bdhB* in the butanoate metabolism pathway (**c**) and *oraE* and *oraS* in the D-arginine and D-ornithine metabolism pathway (**d**) were upregulated in patients with CRC (*n* = 24) than controls (*n* = 24) determined via qPCR with gDNA. Data are presented as the mean ± s.d. of three biological replicates. *P* values were calculated using a two-sided Wilcoxon signed-rank test and were Bonferroni-adjusted. The box plots show the IQRs as boxes, with the median as a black horizontal line and the whiskers extending up to the most extreme points within the 1.5× the IQR. **e**, AUROC matrix of models built with the 175 important EggNOG genes. Values on the diagonal refer to the average AUROC of 20× repeated fivefold stratified cross-validations. Values off the diagonal refer to the AUROCs obtained by training the model on the population of the corresponding row and applying it to the population of the corresponding column. The LOCO row refers to the performances obtained by training the model using all but the cohort dataset of the corresponding column and applying it to the dataset of the corresponding column. The asterisk represents the significance of models assessed with 1,000 permutations (two-sided test). *P = 0.001. Source data

**Extended Data Fig. 8:**
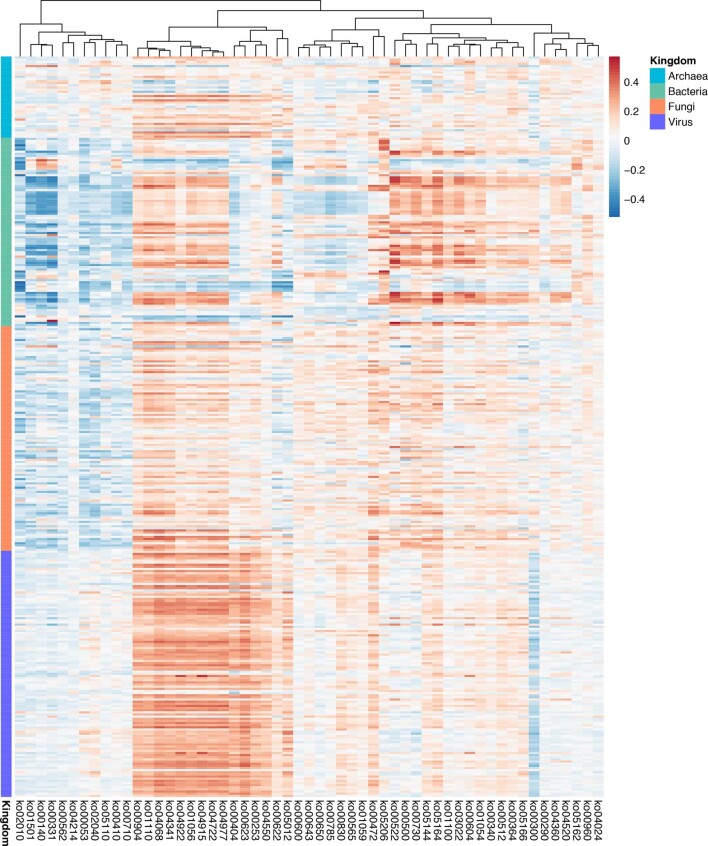
Associations between differential species from four-kingdom and differential functional pathways. Heatmap shows Spearman correlations between bacterial or fungal species and different metabolic pathways as identified with HAlla. Source data

**Extended Data Fig. 9:**
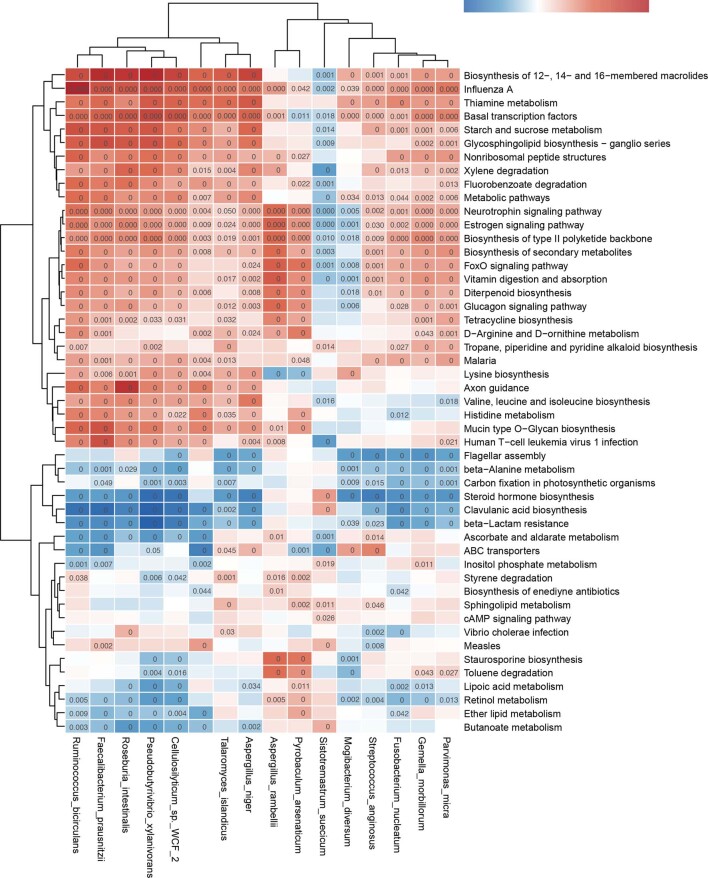
Associations between multi-kingdom markers and differential functional pathways. Heatmap shows Spearman correlations between bacterial or fungal species and different metabolic pathways as identified via HAlla with default parameters. The p value was estimated from two-sided Benjamini-Hochberg-Yekutieli and exact p-value was shown in cells. Source data

For this purpose, we explored the functional alterations at Kyoto Encyclopedia of Genes and Genomes (KEGG) orthology (KO) genes and pathway levels and identified 1,053 differential KO genes, including 612 KO genes with increased abundance in patients with CRC compared to controls (Supplementary Data 10). At the pathway level, we identified 49 differential pathways: 26 were increased and 23 were decreased in patients with CRC, respectively (Supplementary Data 11). Pathways involved in carbohydrate metabolism, such as butanoate, ascorbate and aldarate metabolism, were increased in patients with CRC (Fig. 5a). While D-arginine and D-ornithine metabolism was also enhanced (Fig. 5a), branched-chain amino acids (valine, leucine and isoleucine) and lipid metabolism, such as phospholipase D, were decreased in patients with CRC (Supplementary Data 11). Moreover, associations between these differential pathways and differential species across four kingdoms were identified via HAlla (Supplementary Discussion and Extended Data Figs. 8 and 9).

The data above clearly support the notion that microbiota-mediated functions are altered in CRC, which also relates to multi-kingdom species. We next focused on key genes related to enhanced D-arginine, D-ornithine and butanoate metabolism. The abundance of *bdhA/B* (*P* = 0.0002, *I*^2^ = 0%; Supplementary Data 10) in butanoate metabolism and *oraE* (*P* = 0.004, *I*^2^ = 28.7%; Supplementary Data 10), *oraS* (*P* = 0.009, *I*^2^ = 32.6%; Supplementary Data 10) in D-arginine and D-ornithine metabolism, respectively was significantly increased compared to controls (Fig. 5b), suggesting increased metabolic potentials of aminobutyrate and D-amino acids. Notably, changes in these key genes could be validated in gDNA extracted from the 48 faecal samples of the CHN_SH cohort by exploiting the targeted quantification assay for these genes based on a quantitative PCR (qPCR) protocol developed by Wirbel et al.^13^. The key butanoate metabolism-associated genes, for example, *bdhA* and *bdhB*, were upregulated in patients with CRC (Fig. 5c); D-arginine and D-ornithine metabolism-associated genes, for example, *oraE* and *oraS*, were also more abundant in patients with CRC compared to controls (Fig. 5d).

**Extended Data Fig. 10:**
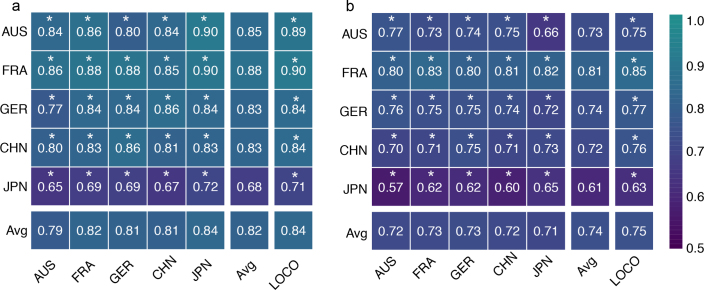
AUROC matrix of models built with the (**a**) 47 important KO genes and (**b**) 20 KEGG pathways. Values on the diagonal refer to the average AUROC of 20-times repeated five-fold stratified cross-validations. Off-diagonal values refer to the AUROCs obtained by training the model on the population of the corresponding row and applying it to the population of the corresponding column. The LOCO row refers to the performances obtained by training the model using all but the cohort dataset of the corresponding column and applying it to the dataset of the corresponding column. Source data

Finally, we assessed the diagnostic capability of differential functions at the EggNOG gene, KO gene and pathway level, respectively. The best predictive accuracy for CRC was achieved by models that were based on 175 EggNOG genes, with an average cross-validation AUROC of 0.86 (Fig. 5e). The average AUROC for models based on differentially expressed KO genes and pathways was 0.82 (Extended Data Fig. 10a) and 0.74 (Extended Data Fig. 10b), respectively. This difference might be rationalized by the fact that individual genes provide more original information than pathways because aggregation of genes into broad functional categories neutralizes variations. Moreover, the gene-based classifier was superior to the species-based classifier, probably due to the greater variability and sensitivity to perturbation of gene-based functional omics^29^.

## Discussion

Most studies have primarily focused on the bacterial microbiota and its effects on human health and disease^12,13,30,31^. Recently, investigations have revealed the critical roles of non-bacterial microorganisms in human diseases^32,33^ (Supplementary Discussion). In this study, we performed a comprehensive analysis on the multi-kingdom microbiome using CRC metagenomic datasets across eight different cohorts. We discovered a series of both bacterial and non-bacterial markers and evaluated their performance in detecting patients with CRC across cohorts. We showed that fungal, archaeal and viral species could separate patients with CRC and healthy controls across multiple geographical cohorts (Fig. 2 and Extended Data Figs. 2–4). However, the predictive value of different kingdom models varied and the bacteria- and fungi-based models, respectively, showed superior accuracy over the archaea- and virus-based models generally (Fig. 2b). Notably, these models showed some preferences at the population scale (Fig. 2b,c), which may be due to differences in geography and lifestyle (Supplementary Discussion). Nevertheless, our findings emphasize the need for integrated analysis to identify universal cross-cohort microbial features for accurate CRC diagnosis.

Previous studies proposed paradigms to identify reproducible microbial biomarkers across multiple datasets and populations by developing machine learning models, followed by cross-study and leave-one-out likely validations^12,13^. Similarly, we developed diagnostic models with multi-kingdom species that significantly improved predictive accuracy (Supplementary Discussion, Fig. 3 and Extended Data Fig. 5). Moreover, models based on the 16-feature panel achieved very high predictive values for CRC diagnosis (average AUROC = 0.83; Fig. 3d), especially early diagnosis (average AUROC = 0.96; Fig. 3e). The panel included some extensively reported bacterial biomarkers (Supplementary Data 7), such as *F. nucleatum*, *P. micra*, *G. morbillorum*, *Pseudobutyrivibrio xylanivorans* and *R. bicirculans*. In addition, fungal species such as *T. islandicus*, *A. rambellii*, *S. suecicum* and *A. niger* were identified as the top 13 important features, highlighting the pivotal roles of non-bacterial microorganisms as diagnostic CRC biomarkers (SupplementaryDiscussion). The association among distinct microbial species may develop into the multi-kingdom ecological drivers of microbiota assembly when adapting to the host microenvironment^34–36^ (SupplementaryDiscussion). However, as yet, the broad cross-species associations during CRC development and progression have not been functionally investigated. It would be interesting to explore whether these associations are merely a bystander effect or contribute to colorectal carcinogenesis.

The functional microbiome is now becoming a prerequisite for host phenotype and physiology and growing efforts have been made to connect the functional traits and mechanisms of organisms to their environments to predict survival, reproduction and community structure^13,37^. It is interesting to note that models based on functional elements also showed good performance in diagnosing CRC (Fig. 5e and Extended Data Fig. 10), especially the EggNOG gene models achieving an average cross-validation AUROC of 0.86, which is even better than species-based models (SupplementaryDiscussion).

In addition, through broad functional metagenomic analysis, we revealed that bacterial–fungal interactions could contribute to CRC pathogenesis via upregulation of D-arginine and D-ornithine and stimulation of the butanoate metabolism pathways. We demonstrated that two marker genes in the D-arginine metabolism pathway, *oraS* and *oraE*, are upregulated in CRC samples compared to controls. Interestingly, the less-studied butanoate metabolism pathway, strongly activated in CRC^38^, was also identified. The CRC driver–passenger model indicates that *F. nucleatum* promotes colorectal tumorigenesis and butanoic acid from the butanoate metabolism pathway plays a critical role in supporting the tumour microenvironment^39^. In line with previous studies^40–42^, we further confirmed a significant enrichment of *bdhA* and *bdhB* in the CRC metagenome. These metabolic disturbances by bacteria, fungi or their associations may indicate the differential host–microbe interactions that could be critical for CRC progression (Supplementary Discussion). Moreover, specific bacterial–fungal interactions are now being explored as a tool to maintain intestinal homoeostasis.

In conclusion, this study presents the most comprehensive metagenomic sequencing-based microbiome study with the largest sample size to date in patients with CRC. We not only systemically explored CRC-associated microbiota, encompassing bacteria, fungi, viruses and archaea, but also identified combined microbial features and provided potential functional insights. Although the application of marker microbes to CRC diagnosis is challenging, especially in asymptomatic individuals, we certainly observed a superior prediction performance of combined multi-kingdoms compared to single kingdoms. Our growing understanding of the role of multi-kingdom microbiomes in patients with CRC could provide hypotheses for the field and inspire investigations into potential applications for CRC diagnosis.

## Methods

### Participant enrollment, informed consent, sample collection and processing of the Chinese cohort

The Chinese cohort in Shanghai (CHN_SH) was recruited to validate the performance of our classification models. Patients were recruited at an initial CRC diagnosis; therefore, patients had not yet received any treatment before their faecal sample collection. Patients with hereditary CRC syndromes or with a previous history of CRC were excluded from the study. Following these criteria, we acquired a cohort of 80 patients with CRC. This study was approved by the Ethics Committee of the School of Life Science of Fudan University and Fudan University Shanghai Cancer Center (ethical approval no. 1809191-7). Healthy controls (86 individuals) with a similar age and sex ratio were selected from the Taizhou Imaging Study (TIS)^43–45^, which is an ongoing longitudinal study intended to explore the aetiology and risk factors of cerebrovascular disease and dementia in three villages that previously showed high response rates from Taixing, China. TIS individuals without physician-diagnosed dementia, stroke, cancer, cardiovascular disease, psychiatric disorders or other serious illnesses were recruited. Written informed consent was obtained from all individuals before data and biospecimen collection. Use of TIS individuals was approved by the Ethics Committee of the School of Life Sciences, Fudan University (institutional review board approval no. 496).

Stool samples were collected in faecal collection tubes and were immediately transferred to a −80 °C freezer until time for use. The gDNA of the faecal specimens was extracted with a Stool Genomic DNA kit (catalogue no. CW2092S; CWBIO) according to the manufacturer’s instructions except for the modification of step 4 with bead-beating for 10 min (Glass beads, acid-washed; catalogue no. G8772; Sigma-Aldrich) to better extract fungal DNA. The details of the DNA extraction method are given in the Supplementary Methods. Sequencing libraries were generated with the NEBNext Ultra DNA Library Prep Kit for Illumina (New England Biolabs) and library quality was confirmed with an Agilent 2100 Bioanalyzer and quantified using real-time PCR. Whole-genome sequencing was carried out on the NovaSeq 6000 system (Illumina). All samples were paired-end sequenced with a 150-base pair (bp) read length to a targeted data set size of 12 Gb. No statistical methods were used to predetermine sample size but our sample sizes are similar to those reported in previous publications^11–13^.

### Public populations of patients with CRC and controls

Raw sequencing data of eight populations from seven countries were downloaded from the Sequence Read Archive (SRA) (details shown in Supplementary Data 1), mainly from two recently published CRC papers^12,13^ and the Japanese cohort^9^. Metadata were manually curated from the published papers.

### Study design

We included a total of 1,368 samples from 9 geographical populations of faecal shotgun metagenomic sequencing data, including publicly available and in-house (CHN_SH) sequencing data. To obtain universal microbial features across different countries, we divided these samples into discovery and validation datasets, with broad regional origin (Supplementary Data 1). Sequencing data of the populations from Austria (AUS, PRJEB7774), France (FRA, PRJEB6070), Germany (GER, PRJEB27928), China (CHN, PRJEB10878) and Japan (JPN, PRJDB4176) were included in our discovery dataset. In total, there were 494 controls and 491 patients with CRC, which included 318 patients with early-stage (stages I and II) and 173 patients with advanced-stage CRC (stages III and IV). The validation dataset consisted of populations from the USA (USA, PRJEB12449), Italy (ITA, SRP136711) and China (CHN_SH, in-house).

### Sequencing data preprocessing

The KneadData (http://huttenhower.sph.harvard.edu/kneaddata) v.0.6 tool was used to ensure data consisting of high-quality microbial reads free from contaminants. Low-quality reads were removed using Trimmomatic (v0.39) (SLIDINGWINDOW:4:20 MINLEN:50 LEADING:3 TRAILING:3). The remaining reads were mapped to the mammalian genome (hg38, felCat8, canFam3, mm10, rn6, susScr3, galGal4 and bosTau8; UCSC Genome Browser) and 21,288 bacterial plasmids (National Center for Biotechnology Information (NCBI) RefSeq database accessed in January 2020), 3,890 complete plastomes (NCBI RefSeq database accessed in January 2020) and 6,093 UniVec sequences (NCBI RefSeq database accessed in January 2020) by bowtie2 v.2.3.5 (ref. ^46^); matching reads that were potentially host-associated and laboratory-associated sequences were removed as contaminant reads.

### Microbial taxonomic and functional profiles

#### Taxonomic profiling

Taxonomic classification of bacteria, archaea, fungi and viruses was assigned to metagenomic reads using Kraken2, an improved metagenomic taxonomy classifier that utilizes *k*-mer-based algorithms^47^. A custom database consisting of 18,756 bacterial, 359 archaeal and 9,346 viral reference genomes from the NCBI RefSeq database (accessed in January 2020) and 1,094 fungal reference genomes from the NCBI RefSeq database (accessed in January 2020), FungiDB (46) (http://fungidb.org) and Ensemble (accessed in January 2020) (http://fungi.ensembl.org) (accessed in January 2020) was built using Jellyfish (v2.3.0) by counting distinct 31-mers in the reference libraries, with each *k*-mer in a read mapped to the lowest common ancestor of all reference genomes with exact *k*-mer matches. Thereafter, each query was classified to a specific taxon with the highest total *k*-mer hits matched by pruning the general taxonomic trees affiliated with the mapped genomes. Bracken (v2.5.0) was used to accurately estimate taxonomic abundance, especially at the species and genus level based on Kraken2 (ref. ^48^). The read counts of species were converted into relative abundance for further analysis.

#### Functional profiling

High-quality reads were preprocessed and assembled into contigs with Megahit v.1.2.9 using ‘meta-sensitive’ parameters; contigs less than 500 bp were discarded from further analysis. Prodigal v.2.6.3 was used to predict genes via the metagenome mode (-p meta). A non-redundant microbial gene reference was constructed with CD-HIT using a sequence identity cut-off of 0.95 and a minimum coverage cut-off of 0.9 for the shorter sequences. The reference was annotated with EggNOG mapper v.2.0.1 based on EggNOG orthology data. Moreover, gene abundance was estimated with CoverM v.0.4.0 (https://github.com/wwood/CoverM) by mapping high-quality reads to reference sequences. An index was created against contigs from the non-redundant genes that originated via the Burrows–Wheeler Aligner (BWA). Clean reads were then mapped to the contig index (BWA MEM) and SAM files were converted into BAM files via SAMtools. Then, CoverM was used to calculate the coverage of genes in the original contigs (coverm contig). The relative abundances of EggNOG genes, KEGG KO groups or pathways were estimated by summing the relative abundances of genes annotated to belong to the same KOs or pathways.

### Integrated analysis to identify differential microbial species and functions

#### Microbial ecological analysis

Alpha diversity metrics, such as Shannon and Simpson Indices of all kingdoms were calculated for each sample. The alpha diversity changes between CRC and control cases were estimated with MaAsLin2 (ref. ^49^), where ‘cohort’ was treated as the fixed effect and body mass index (BMI), sex and age were treated as the random effects. Potential confounding factors with continuous values were transformed into discrete variables either as quartiles, or in the case of BMI into lean (>25), overweight (25–30) and obese (>30) according to conventional cut-offs. In addition, beta diversity was assessed based on Bray–Curtis distance; permutational multivariate analysis of variance (PERMANOVA) was performed to investigate the microbial community differences between disease groups or cohorts with 999 permutations.

#### Differential signature identification

Since microbial profiles are compositional and sparse and heterogeneity exists among different cohorts, MMUPHin^50^ was performed to identify CRC-related differential microbial species, which enables the normalization and combination of multiple microbial community studies. In the MMUPHin analysis, microbial community batch effects among cohorts were corrected with a Combat-like extended method. Microbial profile was arcsine square root-transformed and the age, sex and BMI of individuals were treated as covariates. MMUPHin provides meta-analysis by aggregating individual study results with established fixed effect models to identify consistent overall effects. Species with *P* < 0.05 were identified as differential species and used as candidate features for the CRC diagnosis models. Differential EggNOG gene KOs and pathways were identified as the same pipeline.

### Construction and evaluation of the CRC diagnostic model based on microbial signatures

#### Overview of model construction and evaluation

Based on differential microbial signatures, including multi-kingdom species and multiple functional levels, a comprehensive analysis was performed to investigate potential microbial markers from different dimensions for CRC diagnosis, which mainly included cross-validation model construction and model evaluation, such as cohort-to-cohort, LOCO evaluation and independent validation (Supplementary Fig. 1). To construct a better diagnostic model, we first assessed multiple machine learning algorithms based on our data, such as random forest, neural network and stochastic gradient boosting. The random forest was selected for this study because of its better performance compared to the other approaches in our data (Supplementary Fig. 2) and other studies^11,12^.

#### Feature selection and model construction

For the purpose of distinguishing patients with CRC from healthy controls based on microbial data, we first performed feature selection with the Boruta package (v7.0.0) in R with default parameters (pValue = 0.05, mcAdj = T, maxRuns = 1,000), which iteratively removes features proved by a statistical test to be less relevant than random probes. Correlations between ‘confirmed features’ identified by Boruta were then calculated and only features with a correlation <0.7 were selected to further model construction and avoid colinearity issues. Next, to construct predictive models, we tuned hyperparameters (for example, mtry, ntree, nodesize, maxnodes) using the caret package (v6.0-88). Finally, with the best combination of hyperparameters, we constructed a fivefold cross-validation model to avoid overfitting issues; the model was constructed with each cohort and repeated 20 times. Model significance was accessed with 1,000 permutations with the A3 package (v1.0.0).

#### Generalization of microbial markers

To further test the generalization of CRC microbial markers across technical and geographical differences in multiple populations, we extensively validated the diagnostic models with cohort-to-cohort transfer validation and LOCO validation as described previously^12,13^. Briefly, in cohort-to-cohort transfer validation, the models were trained on a single cohort and their performances were assessed. In LOCO validation, four out of five cohorts in the discovery dataset were pooled as a training set and the remaining cohort was used as an external validation set.

#### Independent validation with external datasets

Furthermore, we used three additional datasets from Italy (ITA), the USA and China (CHN_SH) to perform independent validation analysis and test the robustness of features as CRC diagnostic markers. Like model construction in the discovery cohorts, fivefold cross-validation models were constructed with the identified best panel of multi-kingdom microbial markers and evaluated with the average AUROC. Additionally, we performed cohort-to-cohort and LOCO analyses to further test the robustness of the identified markers. In cohort-to-cohort analysis, models were trained with each cohort in the discovery dataset and tested with each cohort in the validation dataset; in LOCO analysis, models were trained with the combined five cohorts from the discovery dataset and tested with each cohort in the validation dataset.

#### Specificity of microbial markers in non-CRC disease

To avoid false positives in clinical diagnoses, we estimated the specificity of microbial markers for CRC by testing the AUROC values of the models constructed with the best panel of features. These non-CRC diseases included IBD (144 cases and 69 controls from PRJEB1220), T2D (53 cases and 43 controls from PRJEB1786) and PD (31 cases and 28 controls from PRJEB17784).

### Coabundance analysis of multiple kingdoms

To investigate the associations between differential species, FastSpar (v1.0.0)^51^ was performed to construct a compositionality-corrected microbial interactions network capable of estimating correlation values from compositional data. Interactions were calculated with 20 refining interactions, after which the statistical significance of each interaction was estimated within 1,000 permutations. To explore the meta-analysis of coabundance networks in relation to CRC disease, this procedure was performed on each single cohort considering potential heterogeneity among different cohorts; then, we used the Fisher method to combine these independent *P* values in the survcomp package (v1.44.1) and adjusted them with the FDR. Similarly, we calculated the median magnitude of the same interaction partners as the combined association magnitude. Associations with an FDR < 0.00001 were included in the downstream analysis. Network was visualized with Gephi v0.9.2.

### Associations between species and function

Spearman associations between microbial species and their functions were performed using the Hierarchical All-against-All method v.0.8.17 (http://huttenhower.sph.harvard.edu/halla), a computational method used to find multi-resolution associations in high-dimensional, heterogeneous datasets. Associations with an FDR < 0.01 were included in the downstream analysis.

### qPCR of potential CRC-associated genes

To quantify the abundance of the *oraS*, *oraE*, *bdhA* and *bdhB* genes, qPCR as outlined by Wirbel et al.^13^ was performed on a subset of gDNA prepared from randomly selected samples of the CHN_SH cohort (24 controls and 24 patients with CRC). The primers used for validation are listed in Supplementary Data 12 and the patient characteristics are summarized in Supplementary Data 13.

Total microbial DNA was extracted using a Stool Genomic DNA kit according to the manufacturer’s instructions; DNA concentration was determined using NanoDrop. The PCR reactions were prepared with the TB Green Premix Ex Taq II (Tli RNaseH Plus) (catalogue no. RR820A; Takara Bio) containing 0.6 μM of primer and 5 ng of gDNA in a 25 μl final reaction volume. Reactions were performed on a CFX96 Real-Time PCR Detection System (Bio-Rad Laboratories). The cycling programme was set as indicated: initial denaturation at 95 °C for 30 s; 40 cycles of 95 °C for 5 s; 55 °C for 30 s; and 72 °C for 30 s, followed by melting curve analysis.

Gene expression levels were evaluated using the Ct method described previously^13^. Ct values were calculated as the difference between target gene and 16S ribosomal RNA Ct values. *P* values were obtained using a one-tailed Wilcoxon signed-rank test.

### Statistics and reproducibility

No statistical method was used to predetermine sample size since this is an integrated analysis based on public metagenome data with enough samples. No data were excluded from the analyses. The experiments were not randomized because statistical analyses depended on information about cancer status. Data collection and analysis were not performed blind to the conditions of the experiments. Considering microbial data are sparse with a non-normal distribution, relevant statistics were performed with a non-parametric test, such as the Wilcoxon signed-rank test.

### Reporting Summary

Further information on research design is available in the Nature Research Reporting Summary linked to this article.

### Supplementary information

Supplementary Information Supplementary Discussion and Figs. 1 and 2.

Reporting Summary

Supplementary Tables Supplementary datasets 1–13.

### Source data

Source Data Fig. 1 Statistical source data for Fig. 1.

Source Data Fig. 2 Statistical source data for Fig. 2.

Source Data Fig. 3 Statistical source data for Fig. 3.

Source Data Fig. 4 Statistical source data for Fig. 4.

Source Data Fig. 5 Statistical source data for Fig. 5.

Source Data Extended Data Fig. 1 Statistical source data for Extended Data Fig. 1.

Source Data Extended Data Fig. 2 Statistical source data for Extended Data Fig. 2.

Source Data Extended Data Fig. 3 Statistical source data for Extended Data Fig. 3.

Source Data Extended Data Fig. 4 Statistical source data for Extended Data Fig. 4.

Source Data Extended Data Fig. 5 Statistical source data for Extended Data Fig. 5.

Source Data Extended Data Fig. 6 Statistical source data for Extended Data Fig. 6.

Source Data Extended Data Fig. 7 Statistical source data for Extended Data Fig. 7.

Source Data Extended Data Fig. 8 Statistical source data for Extended Data Fig. 8.

Source Data Extended Data Fig. 9 Statistical source data for Extended Data Fig. 9.

Source Data Extended Data Fig. 10 Statistical source data for Extended Data Fig. 10.

## Data Availability

The metagenomic sequencing data of the China-SH validation cohort are deposited in both the NCBI SRA under accession no. PRJNA731589 and the National Omics Data Encyclopedia under accession no. OEP001340. The other raw metagenomic data are available in the SRA (https://www.ncbi.nlm.nih.gov/sra) and European Nucleotide Archive (https://www.ebi.ac.uk/ena/) under accession nos. PRJEB7774, PRJEB10878, PRJEB6070, PRJEB27928, PRJDB4176, PRJEB12449 and PRJNA447983. Source data are provided with this paper.

## Code availability

The code and scripts are available on GitHub (https://github.com/jiaonall/CRC-multi-kingdom.git). The customized code was written in R v.4.0.3.
