# Supplementary material for: Multi-kingdom microbiota analyses identify bacterial–fungal interactions and biomarkers of colorectal cancer across cohorts
Source: Nat Microbiol. 2022 Jan 27;7(2):238–50. doi: 10.1038/s41564-021-01030-7 (PMC8813618; doi:10.1038/s41564-021-01030-7)
Supplement: Supplementary file 1 — Supplementary Discussion and Figs. 1 and 2. [file 41564_2021_1030_MOESM1_ESM.pdf]

nature portfolio

https://doi.org/10.1038/s41564-021-01030-7

Supplementary information

Multi-kingdom microbiota analyses identify bacterial–fungal interactions and biomarkers of colorectal cancer across cohorts

In the format provided by the authors and unedited

Supplementary Information for

Multi-kingdom microbiota analyses identify bacterial-fungal interactions and biomarkers of colorectal cancer across cohorts

Contains

Supplementary Discussion and Supplementary Figures 1-2

Supplementary Discussion

We have analysed the microbial alterations across four kingdoms for CRC samples at phylum level. The abundance of dominant bacteria such as Bacteroidetes was significantly increased (FDR = 0.008, Supplementary Data 3), while that of Firmicutes was decreased (FDR = 0.001, Supplementary Data 3) in CRC patients compared to control. Rare bacterial species such as Fusobacteria (FDR = 0.001, Supplementary Data 3) and Verrucomicrobia (FDR = 0.06, p = 0.006, Supplementary Data 3) were significantly elevated in CRC groups. Regarding the archaea, we observed remarkably increased Euryarchaeota (FDR = 0.00003, Supplementary Data 3) and decreased Crenarchaeota (FDR < 0.00003, Supplementary Data 3) in CRC patients, which accounted for almost 90% of total archaeal reads. Concerning the fungal community, abundance of each phylum showed no significant change between CRC and control subjects (Supplementary Data 3). As for virus, double-strand DNA bacteriophages, such as those of the Myoviridae, Siphoviridae and Podoviridae group, were enriched in both CRC and control groups (the average abundance > 20%), though their abundance also didn't alter significantly. Therefore, our analysis uncovered the multi-kingdom microbial features associated with CRC across different cohorts.

We have determined the CRC-associated microbial species in archaeal and viral kingdom. Among the archaea, the abundances of *Methanobrevibacter smithii*, *Thermococcus kodakarensis* and *Pyrobaculum arsenaticum* were increased in CRC patients across all cohorts (Supplementary Data 4 and Extend Data Fig. 4a). However, the newly identified archaeal species of the TACK group, such as *Sulfuracidifex tepidarius*, *Candidatus Nitrosotenuis cloacae*, were reduced in CRC patients. In addition, the fecal viral kingdom, most of which belong to bacteriophages and have been reported as one of the major components shaping gut microbiota[1,2]. In comparison to control group, we found 115 viral species were increased in CRC patients (Supplementary Data 4 and Extend Data Fig. 4b), such as Lactococcus phage

and Pseudomonas phage, while there were 18 viral species with lower abundances in CRC patients (Supplementary Data 4).

We have examined the diagnostic capability among cohorts. The diagnostic capabilities of all four kingdoms showed great variation across different geographical cohorts. For instance, in the Austrian (AUS) cohort, the bacterial-based model displayed the highest AUROC value, followed by fungi, virus and archaea-based models. In contrast, the AUROCs for the Germany (GER) cohort revealed that the fungal, archaeal and viral-based models were all superior to the bacteria-based model. In addition, compared to other cohorts, the overall AUROC scores of microbe-based models were lower for the Japanese (JPN) population, suggesting distinct gut microbiome characteristics for these CRC patients, most likely due to dietary differences[3].

The Cohort-to-cohort and LOCO analysis on single-kingdom models were performed. Overall, the AUROC scores based on cohort-to-cohort transfer analysis were slightly reduced compared to the above cross-validation models, with an average AUROC of 0.73 for bacterial models, followed by fungal models of 0.72, viral models of 0.69 and archaeal models of 0.67 (Fig. 2c). However, great variability across cohorts could be noted (Fig. 2c). The three western cohorts, AUS, FRA and GER, revealed relatively higher transferability for other cohorts compared to the two eastern cohorts. To reduce the limitations of training on a single dataset, we next performed LOCO analysis. Interestingly, AUROC values were increased compared to those from the cohort-to-cohort transfer analysis (Extended Data Fig. 5a), probably due to the larger size of the “training” dataset. We observed a similar trend for the intra-dataset models with the bacteria-based models achieving the highest average AUROC (0.78), followed by the fungi-based models (average AUROC = 0.74), virus-based models (average AUROC = 0.73) and archaea-based models (average AUROC = 0.70).

We found improved predictability based on combined multi-kingdom features. For example, the cohort-to-cohort transfer AUROCs for our bacteria and archaea-based

models (AB) ranged from 0.60 to 0.84. This represents an average increase of 0.08 ± 0.06 compared to archaea-based models and 0.02 ± 0.06 compared to bacteria-based models. Moreover, the average transfer AUROC for BF models was 0.76, with a comparable AUROC of 0.75 for AB models, which was higher than what any other two-kingdom model achieved (Fig. 3b). Similarly, the prediction accuracy of LOCO analysis was also improved (Extended Data Fig. 6b). Especially the average AUROC of the BF model increased from individual 0.77 (bacteria model), and 0.74 (fungi model), respectively, to 0.79 for the combination.

We further investigated the underlying reasons for addition of more markers which did not further improve model accuracy. Firstly, we found redundant information among features from different kingdoms. After feature selection using the Boruta algorithm, several markers were dropped. The number of markers used for constructing multi-kingdom models is shown in Supplementary Data 5. Notably, the ABF, ABV and ABFV models contained the highest number of markers (41 features).

Secondly, bacterial markers provided the most useful distinguishing capability. All 27 bacterial markers were included in multi-kingdom models (except for 26 bacterial markers in BFV models, Supplementary Data 5), which represented the highest contribution from any kingdom. This is consistent with the notion that single bacteria-based models outperform other single-kingdom models. Therefore, most fungal, archaeal and viral markers were dropped in multi-kingdom models, especially when combined with bacterial markers.

Thirdly, when investigating the models' feature importance, features belonging to bacteria and fungi kingdom generally acted as key features with high rankings in multi-kingdom models. For example, ABFV models with a total 41 features contained 13 bacterial-, five fungal-, one archaeal and one viral marker as top 20 features (Fig. 3c). Collectively, the majority of predictable information was provided by bacterial and fungal markers. Thus, multi-kingdom models would not further enhance the performance of our diagnostic models.

We have analysed the associations between these differential pathways and differential species. In particular, the markers *P. micra*, *P. asaccharolytic*, *E. eligens* from bacteria, as well as *A. rambellii*, *T. islandicus* from fungi were positively associated with D-arginine and D-ornithine metabolism. In contrast, the bacterial markers *R. bicirculans*, *R.intestinalis* as well as the fungal marker *A. niger* were negatively associated with Butanoate metabolism (Extended Data Fig. 11). Furthermore, we also observed the antithetical microbiome-pathways correlations such as the positive correlation between fungi S. suecicum and Butanoate metabolism pathway versus a respectively negative association for the bacterium *P. xylanivorans*. Interestingly, the staurosporine biosynthesis pathway positively correlated with archaeal species P. arsenaticum, similar to *A. rambellii*, but inversely correlated with *P. xylanivorans*, *P. arsenaticum* and *A. rambellii* were shown to be the only two feature species that negatively correlated with lysine biosynthesis.

Previous studies proposed new paradigms to identify reproducible microbial biomarkers across multiple datasets and populations by developing machine learning models, followed by cross-study and leave-one-out-likely validations[4,5]. These are powerful tools to evaluate the robustness and generalizability of identified biomarkers. Similarly, our study discovered a series of potential biomarker species from both bacterial and non-bacterial kingdoms, and evaluated their performance in detecting CRC patients across multiple and distinct populations (Fig. 2). Since striking kingdom preference was found among different populations, we developed diagnostic models with multi-kingdom species using machine learning algorithms, which significantly improved predictive accuracy.

Notably, *A. niger* showed decreased abundance in GER, AUS and FRA cohorts. Furthermore, the abundance of *A. niger* is also decreased at both early and advanced stages compared to healthy controls. *A. niger* reportedly bears anti-cancer properties, e.g. via stimulation of L-asparaginase, which is also used for treating acute myelocytic leukemia, or melanosarcoma[6]. Thus, the observed decreased abundance of

A. niger in CRC patients may be linked to colorectal carcinogenesis. However, this observation awaits further in-depth investigations.

The cross-talk between the microbial metabolome and host could result in metabolic reprogramming of colorectal cells and may not only serve as a risk factor for CRC, but also influence response to therapy[7]. This study revealed bacterial-fungal interactions could contribute to CRC pathogenesis through upregulation of D-arginine and D-ornithine and enrichment of *bdhA* and *bdhB.* For example, the gut commensals can elicit the host immunological responses against extracellular pathogens[8,9]. Moreover, specific fungi-bacterial interactions are now being explored as a tool to maintain intestinal homeostasis. *Saccharomyces boulardii* has been studied extensively to protect against *Clostridium difficile infection through the* protease production. The *Enterobacteriaceae* is required for *C. albicans*-mediated enhancement of colitis[10,11]. These pairwised interactions will be critical when designing therapeutic strategies. However, our metagenomic findings alone does not necessarily represent the transcriptional activity of the D-arginine and butanoate metabolism pathways, respectively, or its relationship to CRC progression. To accurately reflect the CRC microenvironment using above marker genes, additional work will be required to decipher the dynamic transcriptional profile based on RNA from the same cohort and genetic manipulation to confirm their functional role in CRC pathogenesis.

Reference

1. Shkoporov, A.N., *et al.* The Human Gut Virome Is Highly Diverse, Stable, and Individual Specific. *Cell host & microbe* **26**, 527-541.e525 (2019).
2. Gregory, A.C., *et al.* The Gut Virome Database Reveals Age-Dependent Patterns of Virome Diversity in the Human Gut. *Cell host & microbe* **28**, 724-740.e728 (2020).
3. Yachida, S., *et al.* Metagenomic and metabolomic analyses reveal distinct stage-specific phenotypes of the gut microbiota in colorectal cancer. *Nature medicine* **25**, 968-976 (2019).

4. Thomas, A.M., *et al.* Metagenomic analysis of colorectal cancer datasets identifies cross-cohort microbial diagnostic signatures and a link with choline degradation. *Nat. Med.* **25**, 667-678 (2019).
5. Wirbel, J., *et al.* Meta-analysis of fecal metagenomes reveals global microbial signatures that are specific for colorectal cancer. *Nat. Med.* **25**, 679-689 (2019).
6. Nadumane, V.K., Venkatachalam, P. & Gajaraj, B. Aspergillus applications in cancer research. in *New and Future Developments in Microbial Biotechnology and Bioengineering* 243-255 (2016).
7. Jia, W., Xie, G. & Jia, W. Bile acid-microbiota crosstalk in gastrointestinal inflammation and carcinogenesis. *Nature reviews. Gastroenterology & hepatology* **15**, 111-128 (2018).
8. Li, M., *et al.* Microbiota-driven interleukin-17 production provides immune protection against invasive candidiasis. *Crit. Care* **24**, 268 (2020).
9. Shao, T.Y., *et al.* Commensal Candida albicans Positively Calibrates Systemic Th17 Immunological Responses. *Cell Host Microbe* **25**, 404-417 e406 (2019).
10. Sovran, B., *et al.* Enterobacteriaceae are essential for the modulation of colitis severity by fungi. *Microbiome* **6**, 152 (2018).
11. Castagliuolo, I., Riegler, M.F., Valenick, L., LaMont, J.T. & Pothoulakis, C. Saccharomyces boulardii protease inhibits the effects of Clostridium difficile toxins A and B in human colonic mucosa. *Infect. Immun.* **67**, 302-307 (1999).

Supplementary Figures

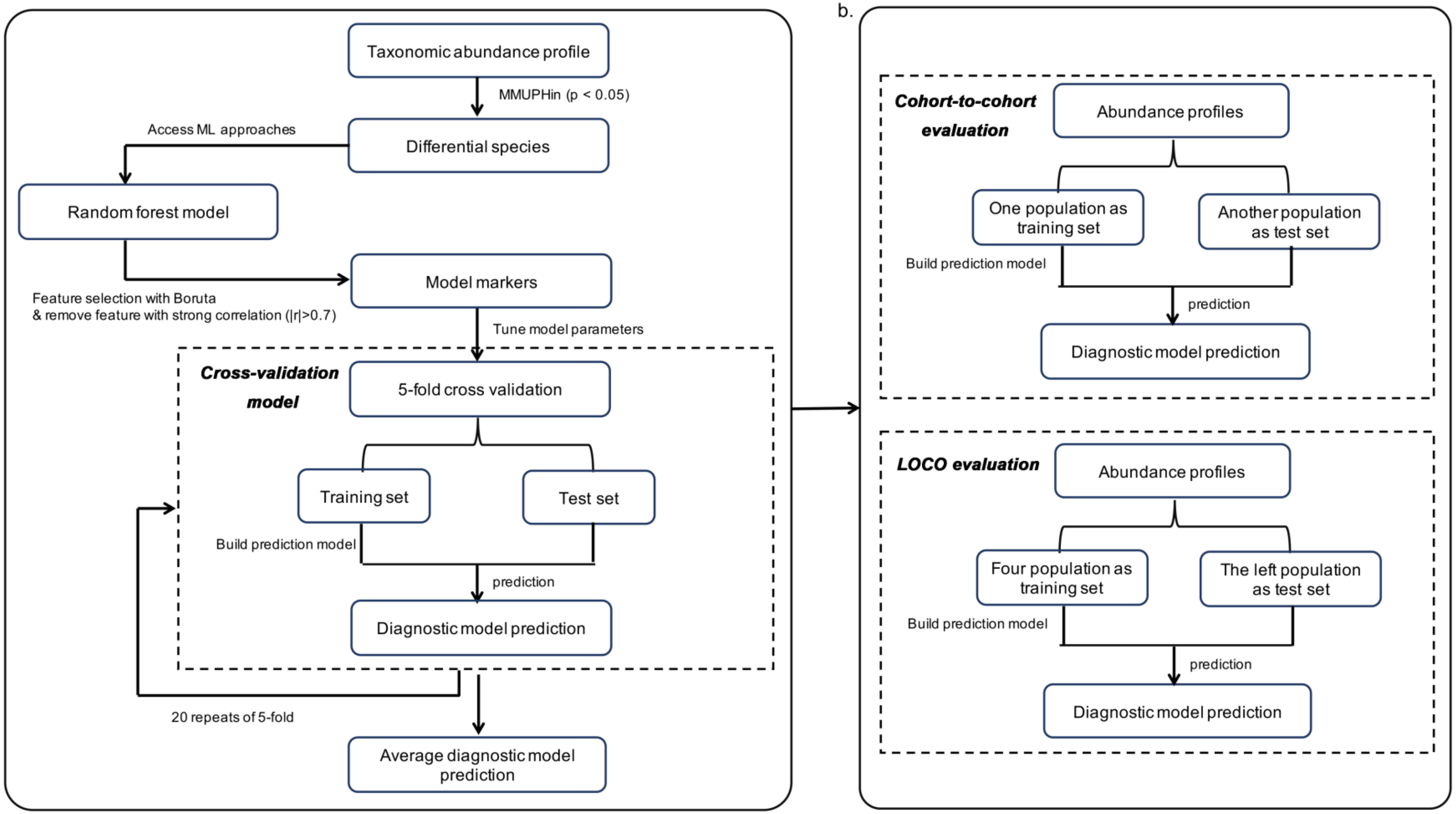

Supplementary Figure 1. The workflow of diagnostic model construction and evaluation. a, The procedure of model constructions mainly included feature selection and cross-validation model construction. In detail, based on the differential species identified by MMUPHin, important features were selected via ‘Boruta’ algorithm. Then five-fold cross-validation random forest models were constructed. **b**, Model/Feature evaluation mainly contains cohort-to-cohort transfer validation and leave-one-cohort-out (LOCO) validation.

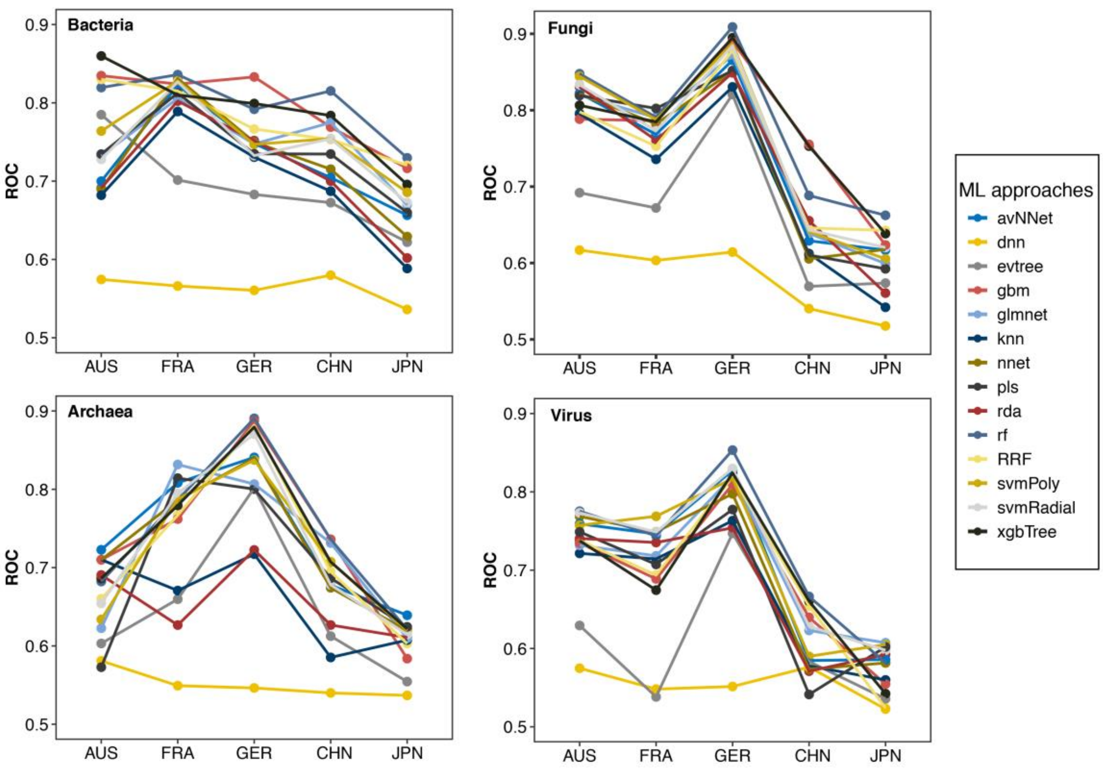

Supplementary Figure 2. Performance of machine learning approaches on multi-kingdom data. While preference of approaches was observed for different datasets, random forest (RF), on average, performed better than other approaches.
