# Supplementary material for: Multi-kingdom microbiota analyses identify bacterial–fungal interactions and biomarkers of colorectal cancer across cohorts
Source: Nat Microbiol. 2022 Jan 27;7(2):238–50. doi: 10.1038/s41564-021-01030-7 (PMC8813618; doi:10.1038/s41564-021-01030-7)
Supplement: Supplementary file 2 — Reporting Summary [file 41564_2021_1030_MOESM2_ESM.pdf]

nature research

Corresponding author(s): Hui Wang, Ruixin Zhu, Guoqing Zhang, Xingdong Chen, Lixin Zhu

Last updated by author(s): Nov 14, 2021

Reporting Summary

Nature Research wishes to improve the reproducibility of the work that we publish. This form provides structure for consistency and transparency in reporting. For further information on Nature Research policies, see our Editorial Policies and the Editorial Policy Checklist.

Statistics

For all statistical analyses, confirm that the following items are present in the figure legend, table legend, main text, or Methods section.

n/a	Confirmed	
☐	☒	The exact sample size (n) for each experimental group/condition, given as a discrete number and unit of measurement
☐	☒	A statement on whether measurements were taken from distinct samples or whether the same sample was measured repeatedly
☐	☒	The statistical test(s) used AND whether they are one- or two-sided<br>*Only common tests should be described solely by name; describe more complex techniques in the Methods section.*
☐	☒	A description of all covariates tested
☐	☒	A description of any assumptions or corrections, such as tests of normality and adjustment for multiple comparisons
☐	☒	A full description of the statistical parameters including central tendency (e.g. means) or other basic estimates (e.g. regression coefficient) AND variation (e.g. standard deviation) or associated estimates of uncertainty (e.g. confidence intervals)
☐	☒	For null hypothesis testing, the test statistic (e.g. F, t, r) with confidence intervals, effect sizes, degrees of freedom and P value noted<br>*Give P values as exact values whenever suitable.*
☒	☐	For Bayesian analysis, information on the choice of priors and Markov chain Monte Carlo settings
☒	☐	For hierarchical and complex designs, identification of the appropriate level for tests and full reporting of outcomes
☐	☒	Estimates of effect sizes (e.g. Cohen's d, Pearson's r), indicating how they were calculated

Our web collection on statistics for biologists contains articles on many of the points above.

Software and code

Policy information about availability of computer code

Data collection	Data was downloaded manually from public databases. For China-SH cohort, the data sequencing was was carried out on the NovaSeq 6000 (Illumina).
Data analysis	KneadData (http://huttenhower.sph.harvard.edu/kneaddata, V.0.6), Trimmomatic (v.0.38), Bowtie2 (v.2.3.5), Megahit (v1.2.9), Prodigal (v2.6.3), EggNOG mapper (v2.0.1), CoverM (v0.4.0, https://github.com/wwood/CoverM)<br>MMUPHin(v.1.8.0), MaAsLin2(v.2.0)<br>HAllA(v.0.8.17)<br>FastSpar(v.1.0.0)

For manuscripts utilizing custom algorithms or software that are central to the research but not yet described in published literature, software must be made available to editors and reviewers. We strongly encourage code deposition in a community repository (e.g. GitHub). See the Nature Research guidelines for submitting code & software for further information.

Data

Policy information about availability of data

All manuscripts must include a data availability statement. This statement should provide the following information, where applicable:
- Accession codes, unique identifiers, or web links for publicly available datasets
- A list of figures that have associated raw data
- A description of any restrictions on data availability

All datasets and raw data generated and/or analyzed during the current study are available from the corresponding author upon reasonable request. The metagenomic sequencing data of the China-SH validation cohort are deposited in the NODE (The National Omics Data Encyclopedia) with accession code OEP001340. Public raw metagenomic data are available in Sequence Read Archive (SRA) (https://www.ncbi.nlm.nih.gov/sra) and European Nucleotide Archive (ENA)

(https://www.ebi.ac.uk/ena/) with the accession IDs PRJEB7774, PRJEB10878, PRJEB6070, PRJEB27928, PRJDB4176, PRJEB12449 and PRJNA447983. The codes and scripts are available on https://github.com/jiaonall/CRC-multi-kingdom. The customized code was written in R 4.0.3.

Field-specific reporting

Please select the one below that is the best fit for your research. If you are not sure, read the appropriate sections before making your selection.

☒ Life sciences ☐ Behavioural & social sciences ☐ Ecological, evolutionary & environmental sciences

For a reference copy of the document with all sections, see nature.com/documents/nr-reporting-summary-flat.pdf

Life sciences study design

All studies must disclose on these points even when the disclosure is negative.

Sample size	This is a study combined 8 population cohorts for a total of 1368 samples. No sample size calculation was performed for this study, all publicly available data sets meeting a minimal set of inclusion criteria were included. New data sets generated (CHN_SH) were of similar sample size as previously published ones that described microbiome alterations in colorectal cancer.
Data exclusions	We used all data from cancer patients and neoplasia-free controls, but did not include any adenoma samples.
Replication	The real-time quantitative PCR experiments were performed for at least three times a week.
Randomization	Not applicable for this observational case-control study.
Blinding	Blinding was not possible because statistical analyses depended on information about cancer status .

Reporting for specific materials, systems and methods

We require information from authors about some types of materials, experimental systems and methods used in many studies. Here, indicate whether each material, system or method listed is relevant to your study. If you are not sure if a list item applies to your research, read the appropriate section before selecting a response.

Materials & experimental systems

n/a	Involved in the study	
☒	☐	Antibodies
☒	☐	Eukaryotic cell lines
☒	☐	Palaeontology and archaeology
☒	☐	Animals and other organisms
☐	☒	Human research participants
☒	☐	Clinical data
☒	☐	Dual use research of concern

Methods

n/a	Involved in the study	
☒	☐	ChIP-seq
☒	☐	Flow cytometry
☒	☐	MRI-based neuroimaging

Human research participants

Policy information about studies involving human research participants

Population characteristics	We report this information in extended data table 1 and figure 1a. 'Cohort' was set as the batch and age, gender and BMI of subjects were treated as covariates.
Recruitment	The Chinese population in Shanghai (CHN_SH) was recruited to validate the performance of classification model. Patients were recruited at initial diagnosis and had not received any treatment before fecal sample collection. Patients with hereditary CRC syndromes, with a previous history of CRC were excluded from the study, while patients with newly diagnosed CRC through colonoscopy were included in the CRC group in this study. Following above criteria, we included 80 CRC patients finally. Healthy controls (86 subjects) with similar age and gender ratio were selected from the Taizhou Imaging Study (TIS).
Ethics oversight	This study was approved by the Ethics Committee of School of Life Science of Fudan University and Fudan University Shanghai Cancer Center, Shanghai, China (Ethical approval number: 1809191-7). Use of TIS subjects was approved by the Ethics Committee of the School of Life Sciences, Fudan University, Shanghai, China (Institutional review board approval number: 496).

Note that full information on the approval of the study protocol must also be provided in the manuscript.
